# Selective Chemistry Enables Simultaneous ImmunoPET and Radioimmunotherapy with a Dual-Labeled Probe

**DOI:** 10.1021/jacsau.6c00555

**Published:** 2026-06-25

**Authors:** Wei-Siang Mark Kao, Camilla Grimaldi, Zachary V. Samuels, Gina Dehlavi, Emilia Strugala, Mike Cornejo, Joni Sebastiano, Lukas M. Carter, Brian M. Zeglis

**Affiliations:** † Department of Chemistry, Hunter College, The City University of New York, New York, New York 10065, United States; ‡ Department of Radiology, 5803 Memorial Sloan Kettering Cancer Center, New York, New York 10065, United States; § Ph.D. Program in Biochemistry, 14772 Graduate Center of City University of New York, New York, New York 10065, United States; ∥ Ph.D. Program in Chemistry, Graduate Center of City University of New York, New York, New York 10065, United States; ⊥ Department of Medical Physics, Memorial Sloan Kettering Cancer Center, New York, New York 10065, United States; # Department of Radiology, Weill Cornell Medical College, New York, New York 10065, United States

**Keywords:** theranostics, radiotheranostics, positron emission tomography, PET, targeted radionuclide therapy, radioimmunotherapy, radiopharmaceutical therapy, zirconium-89, lutetium-177, dual isotope, site-specific bioconjugation, site-selective bioconjugation, strain-promoted azide−alkyne click chemistry, inverse electron-demand Diels–Alder click chemistry, A33, A33 antigen, 5B1, CA19-9, dosimetry

## Abstract

Theranostics is an emerging paradigm in oncology predicated on using nuclear imaging agents to inform and track treatment with radiotherapeutics. Theranostic workflows typically rely upon the administration of two different radiopharmaceuticals, but this approach requires the development of separate probes and depends upon the assumption that the pharmacokinetic profiles of the agents are identical. Herein, we report the creation of a modular platform that enables simultaneous positron emission tomography (PET) and radioimmunotherapy using a vector labeled with two radionuclides. To this end, we synthesized and validated a trifunctional reagent—TzAz-PODS—that facilitates site-selective bioconjugation and allows for the attachment of cargoes via both strain-promoted azide–alkyne and inverse electron-demand Diels–Alder click chemistry. This tool was used to construct an immunoconjugate of the A33 antigen-targeting antibody huA33, which was then labeled in high purity and specific activity with both ^89^Zr and ^177^Lu. ^89^Zr-immunoPET and dual-isotope biodistribution experiments in a murine model of colorectal cancer confirmed the tumor tropism of the radioimmunoconjugate and, more critically, revealed that the ^177^Lu dosimetry derived from the PET data accurately reflected the ^177^Lu dosimetry derived directly from biodistribution studies. Finally, longitudinal radioimmunotherapy studies with the ^89^Zr/^177^Lu-labeled radioimmunoconjugate suggested that PET imaging can help predict response to therapy.

## Graphical abstract

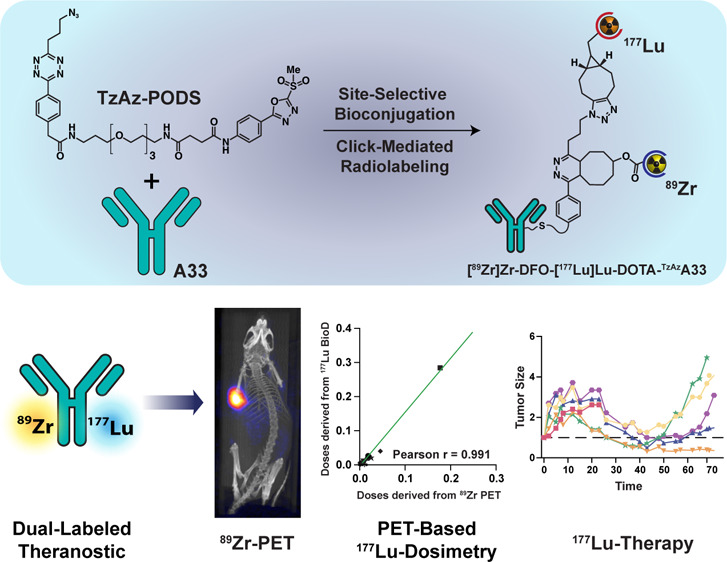

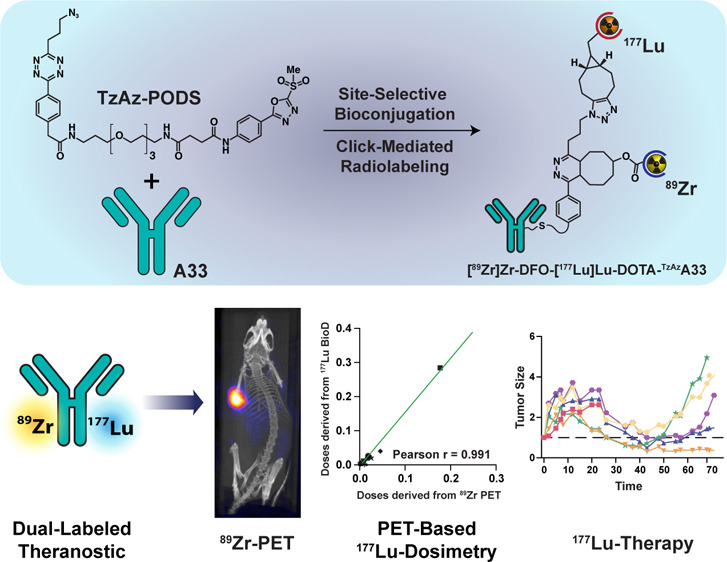

## Introduction

Over the past decade, the principle of theranostics has cemented the place of radiopharmaceuticals in the oncologic mainstream.^1–3^ The concept is simple yet powerful: a radiopharmaceutical labeled with a nuclide suitable for positron emission tomography (PET) or single photon emission computed tomography (SPECT) is used as a companion imaging agent for another probe that has the same molecular target but is labeled with an isotope suitable for therapy. Imaging with the former can be used to identify patients likely to respond to treatment with the latter as well as predict radiation dose rates and track response to therapy.^4–6^ A wide variety of nuclides have been leveraged for radiotheranostics, including isotopes that emit β^+^ (e.g., ^68^Ga, ^18^F, and ^89^Zr) and γ-rays (e.g., ^99m^Tc and ^111^In) for imaging as well as β^–^ (e.g., ^131^I and ^177^Lu) and α-particles (e.g., ^211^At and ^225^Ac) for therapy.

Clinical theranostic workflows typically rely upon the use of two similar yet distinct agents: one for imaging and one for therapy (Figure 1A). This trend is exemplified by well-known theranostics recently approved by the United States Food and Drug Administration and the European Medicines Agency for the imaging and treatment of neuroendocrine tumors (i.e., [^68^Ga]­Ga-DOTA-TATE and [^177^Lu]­Lu-DOTA-TATE)^7–10^ and prostate cancer (i.e., [^68^Ga]­Ga-PSMA-11 and [^177^Lu]­Lu-PSMA-617).^11–13^ There are, of course, a handful of exceptions to this rule. In some cases, different isotopes of the same element can be used for imaging and therapy. While this phenomenon yields theranostic pairs that are chemically identical isotopologues, it is relatively rare and largely limited to radioisotopes of copper (e.g., ^64^Cu and ^67^Cu)^14,15^ and iodine (e.g., ^124^I and ^131^I).^16,17^ In other cases, a single radionuclide is capable of producing emissions that are suitable for both imaging and therapy. This means a single agent could be leveraged for both sides of the theranostic divide, but such isotopes are either rare (e.g., ^161^Tb)^18,19^ or exhibit suboptimal properties for imaging (e.g., ^177^Lu)^20–23^ or therapy (e.g., ^64^Cu).^14,15,24^ Special cases aside, the overwhelming majority of radiotheranostic pairs are labeled with two different nuclides. While this has undeniably proven effective, it is not without challenges. To wit, this approach necessitates the development and validation of two separate probes and depends upon the assumption that the pharmacokinetic profiles of the agents are identical. The former is expensive and labor intensive; the latter is not always a reasonable supposition, as changes in chelator and radiometal have been shown to dramatically influence the pharmacokinetic profile of radiotracers.

**Figure 1:**
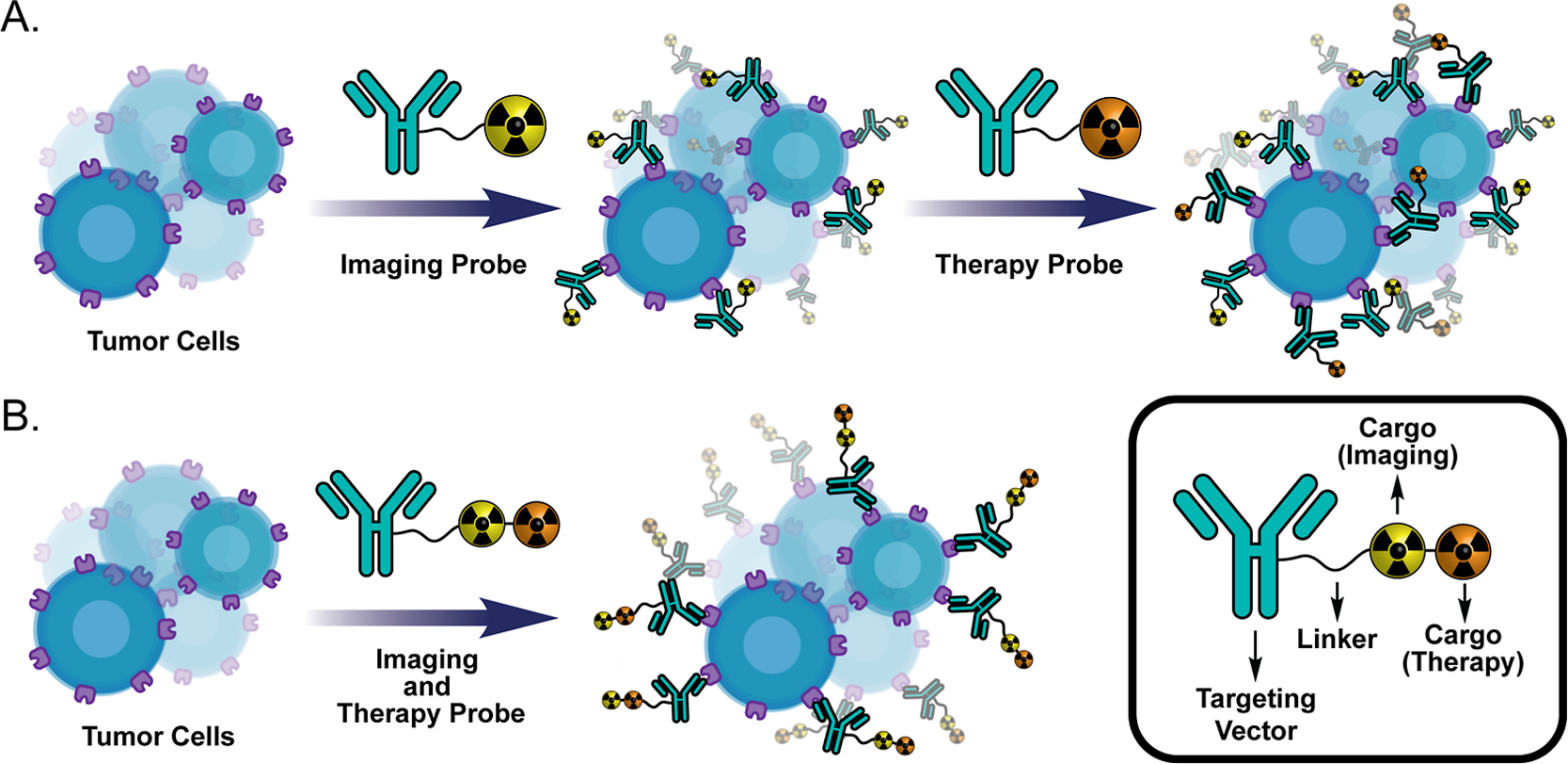
(A) Schematic of a traditional radiotheranostic workflow in which two radiopharmaceuticals—one for imaging and one for therapy—are used; (B) schematic of the radiotheranostic paradigm introduced by this work, in which a single, dual-labeled agent is used for imaging and therapy.

To provide an alternative to this paradigm, we have developed a modular platform for the creation of radiotheranostics labeled with two different nuclides (Figure 1B). Such dual-labeled probes would enable simultaneous PET and therapy. Critically, the imaging data could provide real-time insight into the dosimetry of the therapeutic nuclide and could thus guide subsequent doses of the radiotherapeutic. Moreover, the imaging results could help predict the response of patients to therapy, further aiding in the proactive management of the disease. As a proof of concept, we sought to label the A33 antigen-targeting antibody huA33 with both the β^+^-emitting radiometal ^89^Zr (*t*_1/2_ ∼ 3.3 d) and the β^–^-emitting radiolanthanide ^177^Lu (*t*_1/2_ ∼ 6.8 d) and then validate the imaging and therapeutic performance of the radioimmunoconjugate in a murine model of colorectal cancer.

The radiolabeling of an antibody with two different radioactive cargoes poses two technical challenges. First, the modification of an antibody with any cargo (let alone two) risks impairing its ability to bind its antigen as well as altering its physiochemical properties. Second, the two radiometals in question are best coordinated by different chelators—desferrioxamine (DFO) for ^89^Zr^4+^ and 1,4,7,10-tetraazacyclododecane-1,4,7,10-tetraacetic acid (DOTA) for ^177^Lu^3+^—but each is able to bind that of the other, albeit with lower affinity and stability.^25^ This means that we cannot simply label a DFO- and DOTA-bearing immunoconjugate with both ^89^Zr^4+^ and ^177^Lu^3+^, as this would risk having some fraction of each radiometal bound to the “wrong” chelator, a situation that could result in the inadvertent release of free radiometals within the body.

Selective chemistries were leveraged to address both challenges. With respect to the former, we turned to a site-selective approach to bioconjugation predicated on the irreversible ligation between phenyloxadiazolyl methyl sulfone (PODS) reagents and free sulfhydryl groups created via the reduction of the immunoglobulin’s interchain disulfides. We have previously shown that PODS reagents facilitate the synthesis of stable, well-defined, and homogeneous radioimmunoconjugates that boast superior in vivo performance compared to analogous agents created via traditional, stochastic bioconjugation methods.^26,27^ With regard to the second, we have harnessed two different types of click chemistry to facilitate the stepwise assembly of the dual-labeled radioimmunoconjugate: the strain-promoted azide-alkyne cycloaddition (SPAAC) reaction and the inverse electron-demand Diels-Alder (IEDDA) reaction. Both bioorthogonal transformations have been used to significant effect in recent years for the efficient and modular construction of radiopharmaceuticals.^28,29^ Once the aforementioned technologies were leveraged to create a novel ^89^Zr- and ^177^Lu-labeled antibody, the in vivo performance of the radioimmunoconjugate was interrogated via immunoPET, biodistribution, and longitudinal therapy studies in a murine model of colorectal cancer.

While several targeted probes labeled with two different cargoes—e.g., a radionuclide and a toxin or a fluorophore and a radionuclide—have been described in the literature, this work represents, to the best of our knowledge, the first in vivo evaluation of a dual-labeled radiopharmaceutical. That said, a handful of other papers have explored the labeling of radiopharmaceuticals with two different radionuclides.^31–34^ In 2018, for example, Wurzer et al. leveraged click chemistry and the DOPTI and TRAP chelators to create a PSMA-targeted radiopharmaceutical that could be labeled with both ^68^Ga and ^213^Bi, though their in vitro and in vivo characterization experiments were limited to probes labeled with only one of the two radionuclides.^32^ More recently, Xu et al. developed ^125^I- and ^111^In-labeled caspase-3-targeted probes that enabled simultaneous, quantitatively separable SPECT imaging (but not therapy).^34^ Finally, very recent developments with respect to the use of CHX-A″-DTPA for the coordination of ^89^Zr^4+^ offers a potential route to single probes labeled with ^177^Lu^3+^ and ^89^Zr^4+^, but the use of such a system is (obviously) limited to these two isotopes.^35^

## Results and Discussion

### Synthesis and Bioconjugation

The nexus of our approach to the synthesis of dual-labeled radioimmunoconjugates is TzAz-PODS, a trifunctional molecule that enables the site-selective modification of the immunoglobulin via the irreversible modification of cysteine residues as well as the attachment of two different cargoes via a pair of bioorthogonal click ligations: the SPAAC and IEDDA reactions. TzAz-PODS was synthesized in a convergent manner via the coupling of a carboxylic acid-bearing variant of TzAz (i.e., TzAz-COOH) and an amine-bearing variant of PODS (i.e., PODS-NH_2_). TzAz-COOH was synthesized from 4-bromobutyronitrile in three steps in 86% yield, and PODS-NH_2_ was synthesized in four steps from 5-(4-aminophenyl)-1,3,4-oxadiazole-2-thiol in 70–85% yield. The two components were then coupled together to yield the final product, TzAz-PODS, in 60–75% overall yield (*n* = 3) and >95% purity. The final product as well as all intermediates were purified using flash silica chromatography or reverse-phase C_18_ HPLC and characterized via ^1^H NMR, ^13^C NMR, and mass spectrometry (Schemes S1–S3 and Figures S19–S26).

We next performed the site-selective bioconjugation of the A33 antigen-targeting antibody A33. To this end, we reduced the interchain disulfides of the immunoglobulin using tris­(2-carboxyethyl)­phosphine (TCEP), incubated the product with 10 equiv of TzAz-PODS for 1 h at 37 °C, and purified the resultant immunoconjugate—^TzAz^A33—via PD-10 gel filtration chromatography. Size-exclusion chromatography confirmed the purity of the immunoconjugate, and MALDI-ToF mass spectrometry revealed a degree of labeling (DOL) of 2.3 ± 0.2 TzAz/mAb (Tables S1 and S2). As an additional proof-of-concept, the bioconjugation reaction was also performed with the CA19-9-targeting mAb 5B1 to produce ^TzAz^5B1, and a similar DOL was obtained: 2.3 ± 0.1 TzAz/mAb (Tables S1 and S2).

With the site-selectively modified immunoconjugates in hand, we next turned to the attachment of cargoes. To this end, ^TzAz^A33 was incubated with either a *trans*-cyclooctene-bearing variant of Cy5 (TCO-Cy5), a bicyclo[6.1.0]­nonyne-modified variant of AlexaFluor 488 (BCN-AF488), or both (Figure 2B). The attachment of the fluorophores was verified via SDS-PAGE, and both UV–vis spectrophotometry and MALDI-ToF mass spectrometry were used to ascertain that the DOL for each immunoconjugate was ∼2 Cy5/mAb and/or ∼2 AlexaFluor 488/mAb (Figure S2 and Table S2). Subsequently, the fluorophore-bearing immunoconjugates were used for immunocytochemistry (ICC) with A33 antigen-expressing SW1222 human colorectal cancer cells (Figures 2C and S4). These cells were chosen as the model system for this study because the in vitro and in vivo behavior of A33-based immunoconjugates with SW1222 cells and xenografts is well understood, facilitating comparisons between the data obtained with these dual-labeled probes and previous work. The ICC results revealed that Cy5-^TzAz^A33, AF488-^TzAz^A33, and Cy5/AF488-^TzAz^A33 all stained the antigen-positive cells effectively and that the signals from the two fluorophores colocalized to a high degree in all cases (>80%). To illustrate the modularity of the platform, another immunoconjugate—^TzAz^5B1—was similarly modified with either TCO-Cy5, a dibenzocyclooctyne-bearing variant of IR800 (DBCO-IR800), or both (Figure S2). The attachment of the fluorophores was verified via SDS-PAGE, UV–vis spectrophotometry, and MALDI-ToF mass spectrometry, which revealed DOLs of ∼2 Cy5/mAb and/or ∼2 IR800/mAb, and immunocytochemistry with CA19-9-expressing BxPC3 human pancreatic ductal adenocarcinoma cells underscored the efficacy of the novel immunoconjugates (Figure S3).

**Figure 2:**
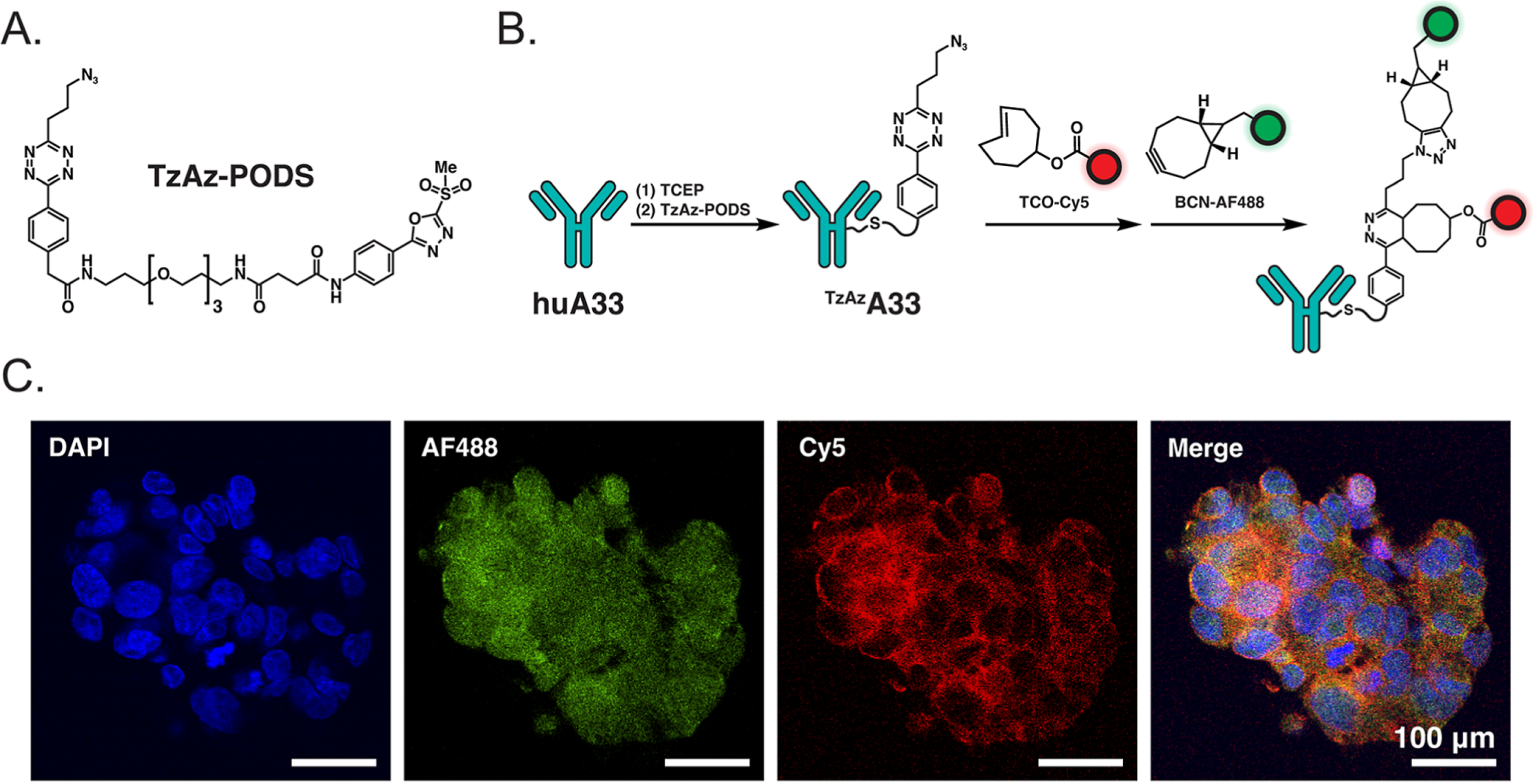
(A) Structure of TzAz-PODS; (B) schematic of the synthesis of Cy5/AF488-^TzAz^A33; (C) immunocytochemical staining of A33 antigen-expressing SW1222 human colorectal cancer cells with Cy5/AF488-^TzAz^A33 depicting DAPI (blue), AF488 (green), Cy5 (red), and merged signals.

### Radiochemistry

We chose to use the A33 antibody as the model system for the radiochemical and in vivo validation studies. Three different radioimmunoconjugates were synthesized to probe the degree to which the bioconjugation and chelation architectures affect the in vivo performance of the probes: [^89^Zr]­Zr-DFO-^TzAz^A33, [^89^Zr]­Zr-DFO-DOTA-^TzAz^A33, and [^89^Zr]­Zr-DFO-[^177^Lu]­Lu-DOTA-^TzAz^A33 (Figure 3). For the first, ^TzAz^A33 was reacted with a TCO-bearing variant of DFO (TCO-DFO), purified via gel filtration chromatography, and then incubated with [^89^Zr]­Zr^4+^ to yield [^89^Zr]­Zr-DFO-^TzAz^A33 in high radiochemical yield (>95%), radiochemical purity (>99%), and specific activity (3 mCi/mg). For the second, [^89^Zr]­Zr-DFO-^TzAz^A33 was reacted with BCN-bearing derivative of DOTA (BCN-DOTA) and subsequently purified via gel filtration to yield [^89^Zr]­Zr-DFO-DOTA-^TzAz^A33 in equally high yield, purity, and specific activity. Finally, for the third, BCN-DOTA was radiolabeled with [^177^Lu]­Lu^3+^ in a molar activity of 10.0 mCi/μmol and used after purification to modify [^89^Zr]­Zr-DFO-^TzAz^A33 (Scheme S4). The resultant radioimmunoconjugate—[^89^Zr]­Zr-DFO-[^177^Lu]­Lu-DOTA-^TzAz^A33—was obtained in >95% radiochemical yield and >99% purity with specific activities of ∼3.0 mCi/mg (^89^Zr) and ∼3.0–6.0 mCi/mg (^177^Lu) after purification (Table S3 and Figure S5). Radio-instant thin-layer chromatography experiments confirmed that all three radioimmunoconjugates remained >75% stable to demetalation after incubation in human serum for 5 d at 37 °C (Figure S5). Furthermore, radio-size-exclusion high-performance liquid chromatography (radio-SE-HPLC) demonstrated that all the three probes were similarly stable to demetalation, fragmentation, and aggregation after incubation in PBS (pH 7.4) for 5 d at 37 °C (Figures S6–S11). Immunoreactivity measurements with magnetic beads coated with recombinant A33 antigen illustrated that the radioimmunoconjugates had immunoreactive fractions of >0.7 ([^89^Zr]­Zr-DFO-^TzAz^A33), >0.9 ([^89^Zr]­Zr-DFO-DOTA-^TzAz^A33), and >0.9 [^89^Zr]­Zr-DFO-[^177^Lu]­Lu-DOTA-^TzAz^A33 (Figure S5) and that the binding of each of the agents to the antigen could be blocked with excess unlabeled probe. Finally, complementary ELISA measurements using parental A33 as well as the unlabeled immunoconjugates—i.e. ^TzAz^A33, DFO-^TzAz^A33, and DFO-DOTA-^TzAz^A33—confirmed that the bioconjugation process did not impair the ability of the immunoconjugates to bind their antigen (Figure S12).

**Figure 3:**
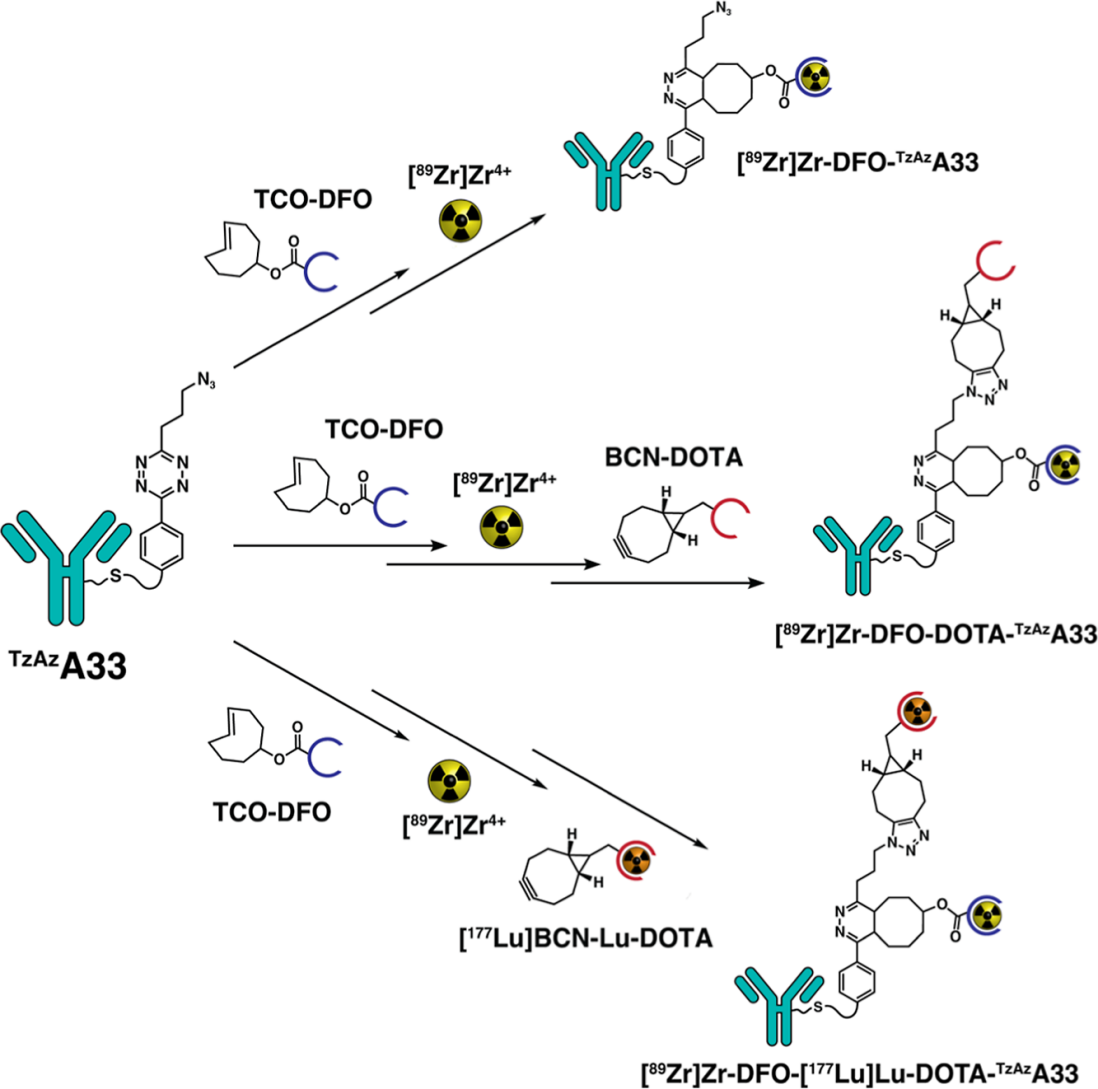
Schematics for the synthesis of [^89^Zr]­Zr-DFO-^TzAz^A33, [^89^Zr]­Zr-DFO-DOTA-^TzAz^A33, and [^89^Zr]­Zr-DFO-[^177^Lu]­Lu-DOTA-^TzAz^A33.

### Pilot In Vivo Experiments

PET/CT imaging and biodistribution studies were performed to evaluate the in vivo performance of these radioimmunoconjugates in a murine model of colorectal cancer. To this end, athymic nude mice bearing subcutaneous SW1222 xenografts (*n* = 4 per group) were injected with [^89^Zr]­Zr-DFO-^TzAz^A33, [^89^Zr]­Zr-DFO-DOTA-^TzAz^A33, or [^89^Zr]­Zr-DFO-[^177^Lu]­Lu-DOTA-^TzAz^A33 (100 μCi ^89^Zr in 100 μL of 0.9% sterile saline) via the lateral tail vein. PET data collected 24, 72, and 120 h postinjection revealed that all three radioimmunoconjugates exhibited very similar pharmacokinetic profiles (Figures 4A and S13–S15). In each case, uptake in the tumor was clear by 24 h p.i. and increased dramatically over the course of the experiment, while very little accretion was observed in any healthy tissues. Biodistribution data acquired after the terminal imaging time point reinforced the imaging data, revealing ^89^Zr activity concentrations <5%ID/g in most healthy tissues and similar tumoral uptake values for [^89^Zr]­Zr-DFO-^TzAz^A33 (27.6 ± 11.5%ID/g), [^89^Zr]­Zr-DFO-DOTA-^TzAz^A33 (26.4 ± 8.9%ID/g), and [^89^Zr]­Zr-DFO-[^177^Lu]­Lu-DOTA-^TzAz^A33 (38.7 ± 14.1%ID/g) (Figure 4B and Table S4). Critically, the ^89^Zr and ^177^Lu counts were measured separately during the biodistribution experiment using different voltage channels on the gamma counter, and the activity concentrations of both nuclides were nearly indistinguishable in all tissues (Figure 4C and Table S5). Indeed, the only statistically significant difference observed was in the tumor—^89^Zr = 38.7 ± 14.1%ID/g and ^177^Lu = 28.4 ± 6.1%ID/g, *p* = 0.02—and this discrepancy was not seen in subsequent experiments (vide infra). Taken together these data underscore two critical points: (1) the presence of DOTA and ^177^Lu did not dramatically impact the pharmacokinetic profile of the radioimmunoconjugates and (2) [^89^Zr]­Zr-DFO-[^177^Lu]­Lu-DOTA-^TzAz^A33 produced nearly identical ^89^Zr and ^177^Lu biodistribution data.

**Figure 4:**
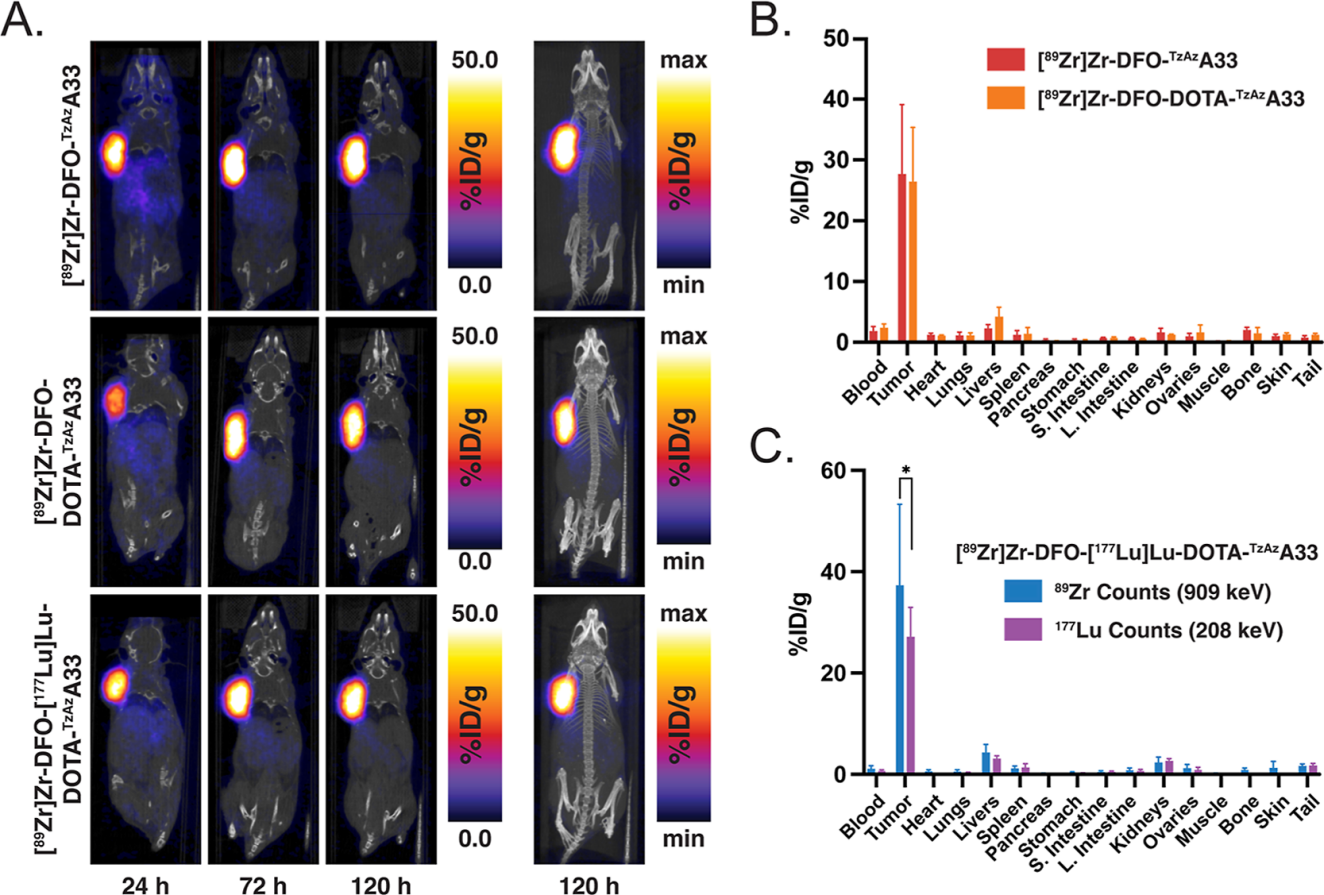
(A) Coronal PET/CT images acquired 24, 72, and 120 h after the administration of [^89^Zr]­Zr-DFO-^TzAz^A33, [^89^Zr]­Zr-DFO-DOTA-^TzAz^A33, and [^89^Zr]­Zr-DFO-[^177^Lu]­Lu-DOTA-^TzAz^A33 to athymic nude mice bearing SW1222 tumors; maximum intensity projection images acquired 120 h after the administration of the radioimmunoconjugates to the same tumor-bearing mice; (B) ^89^Zr biodistribution data acquired 120 h after the administration of [^89^Zr]­Zr-DFO-^TzAz^A33 and [^89^Zr]­Zr-DFO-DOTA-^TzAz^A33; (C) ^89^Zr and ^177^Lu biodistribution obtained on separate channels (909 and 208 keV, respectively) 120 h after the administration of [^89^Zr]­Zr-DFO-[^177^Lu]­Lu-DOTA-^TzAz^A33.

### Correlations between PET Imaging and Dosimetry

These initial in vivo experiments prompted the next question in the investigation: whether immunoPET with the ^89^Zr/^177^Lu-labeled radioimmunoconjugate can provide a readout of the compound’s ^177^Lu dosimetry. More precisely, we sought to determine whether dosimetry data derived from PET images collected following the administration of [^89^Zr]­Zr-DFO-[^177^Lu]­Lu-DOTA-^TzAz^A33 aligned with ^177^Lu dosimetry data derived directly from ex vivo biodistribution data. To address this question, 8 cohorts of athymic nude mice bearing subcutaneous SW1222 xenografts (*n* = 4 per cohort) were administered [^89^Zr]­Zr-DFO-[^177^Lu]­Lu-DOTA-^TzAz^A33 (100–110 μCi ^89^Zr; 100–110 μCi ^177^Lu; ∼33 μg; 100 μL 0.9% sterile saline). One cohort of mice was imaged after 24, 48, 72, 96, 120, 144, and 168 h, while the other seven were sacrificed for biodistribution data 4, 24, 48, 72, 96, 120, 144, and 168 h after injection (Figures 5A,B, S16,S17, and Tables S6,S7). These imaging and biodistribution data reinforced several observations made during the pilot experiments: (i) the ^89^Zr/^177^Lu-labeled radioimmunoconjugate accumulated in tumor tissue and was retained throughout the time course of the experiment, (ii) healthy tissues displayed lower levels of uptake that decreased over the course of the experiment, and (iii) the ^89^Zr and ^177^Lu biodistribution data aligned very closely. The only organ with substantially different ^89^Zr and ^177^Lu activity concentration values across time points was the bone, presumably due to the in vivo release of small amounts of the osteophilic radiometal ^89^Zr^4+^ from the radioimmunoconjugate (while free ^177^Lu^3+^ can also accumulate in the bone, it does not typically exhibit bone-seeking behavior comparable to that of ^89^Zr^4+^).

**Figure 5:**
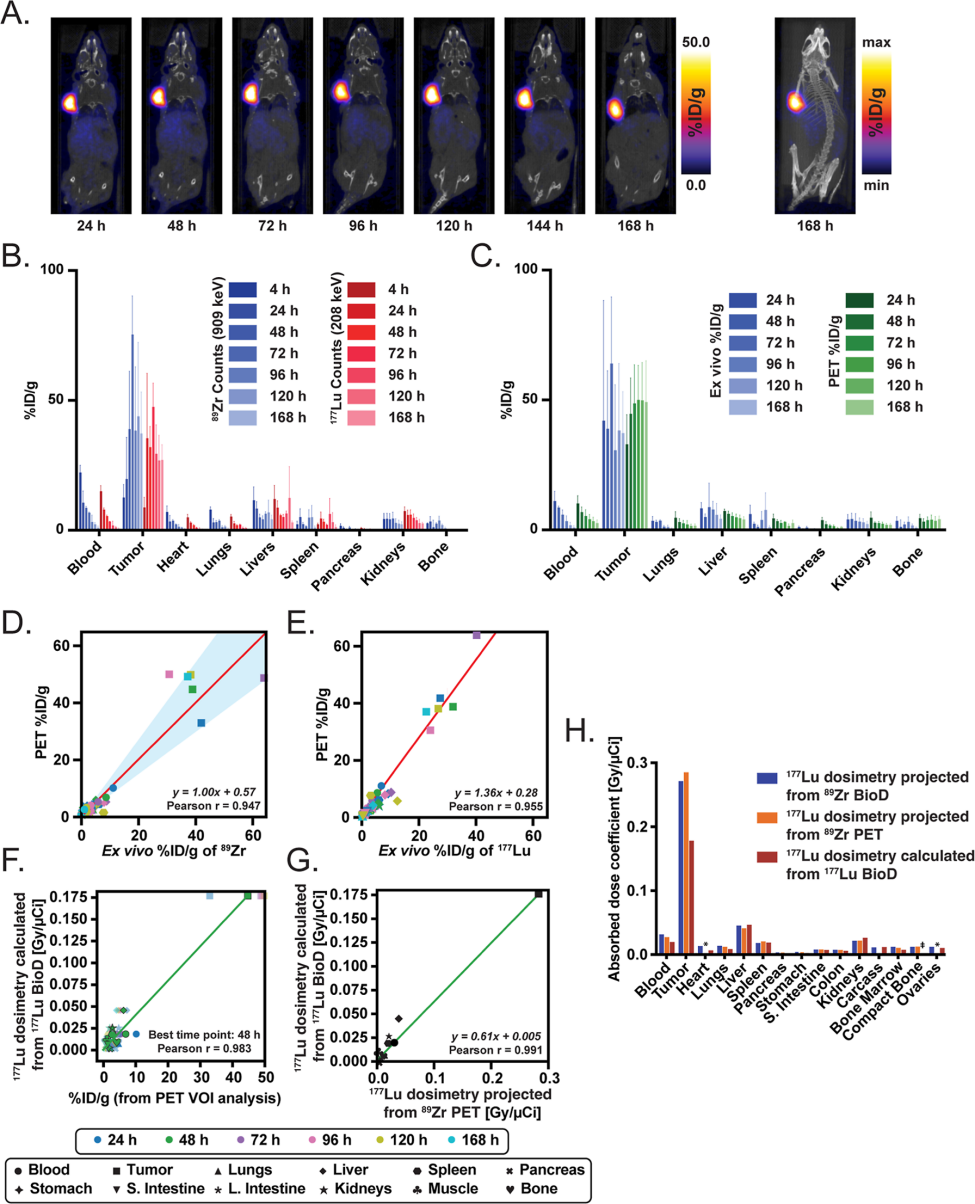
(A) Coronal (left) and MIP (right) PET images acquired following the administration of [^89^Zr]­Zr-DFO-[^177^Lu]­Lu-DOTA-^TzAz^A33 to athymic nude mice bearing subcutaneous SW1222 xenografts; (B) ex vivo ^89^Zr and ^177^Lu distribution data obtained 4, 24, 48, 72, 96, 120, 144, and 168 h after the administration of the ^89^Zr/^177^Lu-labeled radioimmunoconjugate; (C) PET- and ex vivo biodistribution-derived ^89^Zr activity concentration data; (D) PET-derived vs biodistribution-derived ^89^Zr activity concentrations; (E) PET-derived activity concentrations vs biodistribution-derived ^177^Lu activity concentrations; (F) comparison of ^177^Lu dose coefficients (Gy/μCi) derived from longitudinal ^177^Lu biodistribution data and ^89^Zr PET-derived activity concentrations at individual time points; (G) comparison of ^177^Lu dose coefficients (Gy/μCi) derived from longitudinal ^177^Lu biodistribution data and ^177^Lu dose coefficients (Gy/μCi) projected using serial ^89^Zr PET data; (H) comparison of ^177^Lu dose coefficients calculated based on ^177^Lu biodistribution data (red), ^89^Zr biodistribution data (blue) and ^89^Zr PET data (orange) (*Below the limit of detection; ^‡^no VOI analyzed).

To determine whether PET imaging alone can reliably estimate tissue activity concentrations, %ID/g values were extracted from PET volumes of interest (VOIs) and subsequently leveraged for dosimetry calculations using methods previously reported by Carter and Zanzonico.^30^ Briefly, CT-guided volumes of interests were defined for each organ and used for PET image analysis. For larger organs, spherical VOIs were placed centrally and at least one value of full width at half-maximum (fwhm = ∼2 mm) away from the organ boundaries whenever possible, and then the mean activity was measured. For small regions—i.e. <5 mm in any dimension—VOIs were manually contoured using CT guidance, and the maximum activity within the VOI was used to quantify uptake. Uptake values were decay-corrected and reported as %ID/g unless otherwise stated. These values were compared directly with ex vivo ^89^Zr biodistribution data, and these two sets of uptake values demonstrated strong concordance across organs and time points, with most data points falling within a slope range of 0.8–1.2 relative to the line of identity (Figure 5C,D). This clearly indicates minimal quantitative bias in the PET measurements. Furthermore, strong concordance was observed between the PET-derived ^89^Zr activity concentrations and the biodistribution-derived ^177^Lu activity concentrations (Pearson *r* value = 0.955; Figure 5E). Shifting to dosimetry, the dose coefficients (Gy/μCi) derived from the longitudinal ^177^Lu biodistribution data exhibited strong correlations with the ^89^Zr-PET-derived activity concentrations measured at individual time points, with measurements taken 48 h p.i. providing the strongest concordance (Pearson *r* value = 0.983; Figure 5F). Finally—and most importantly—the ^177^Lu dose coefficients derived from the longitudinal ^177^Lu biodistribution data aligned very closely with the ^177^Lu dose coefficients projected using the serial ^89^Zr-PET data (Pearson *r* value = 0.991; Figure 5G,H; Table S10). Taken together, these data indicate that quantitative ^89^Zr-PET measurements reliably reflect the in vivo distribution of the therapeutic radionuclide and can accurately predict ^177^Lu-based absorbed doses, supporting the use of ^89^Zr PET as a readout of the therapeutic dosimetry of [^89^Zr]­Zr-DFO-[^177^Lu]­Lu-DOTA-^TzAz^A33.

### Pairing PET with Longitudinal Therapy

A longitudinal therapy study was conducted to evaluate the theranostic potential and therapeutic efficacy of the dual-labeled radioimmunoconjugate. To this end, athymic nude mice bearing subcutaneous SW1222 xenografts (*n* = 6) were administered [^89^Zr]­Zr-DFO-[^177^Lu]­Lu-DOTA-^TzAz^A33 (100 μCi ^89^Zr, 200 μCi ^177^Lu, 33.3 μg, in 100 μL 0.9% sterile saline) via the lateral tail vein. Over the course of the experiment, tumor volumes and body weights were measured every 3 d, and blood was collected every 7 d via the retro-orbital sinus for hematoxicological analyses. ^89^Zr-immunoPET images were collected 48 h postinjection—i.e., the time point that provided the strongest correlation between the ^89^Zr-PET- and ^177^Lu-biodistribution-derived ^177^Lu dosimetry (see Figure 5E)—and volumes of interest were drawn over tumor tissue to derive tumoral activity concentrations (Figure 6A). These numbers were used to extract ^89^Zr dosimetry data (Figures S18) which were in turn used to estimate ^177^Lu dosimetry in individual mice based on the ratio of ^89^Zr and ^177^Lu activities (i.e., 1:2).

**Figure 6:**
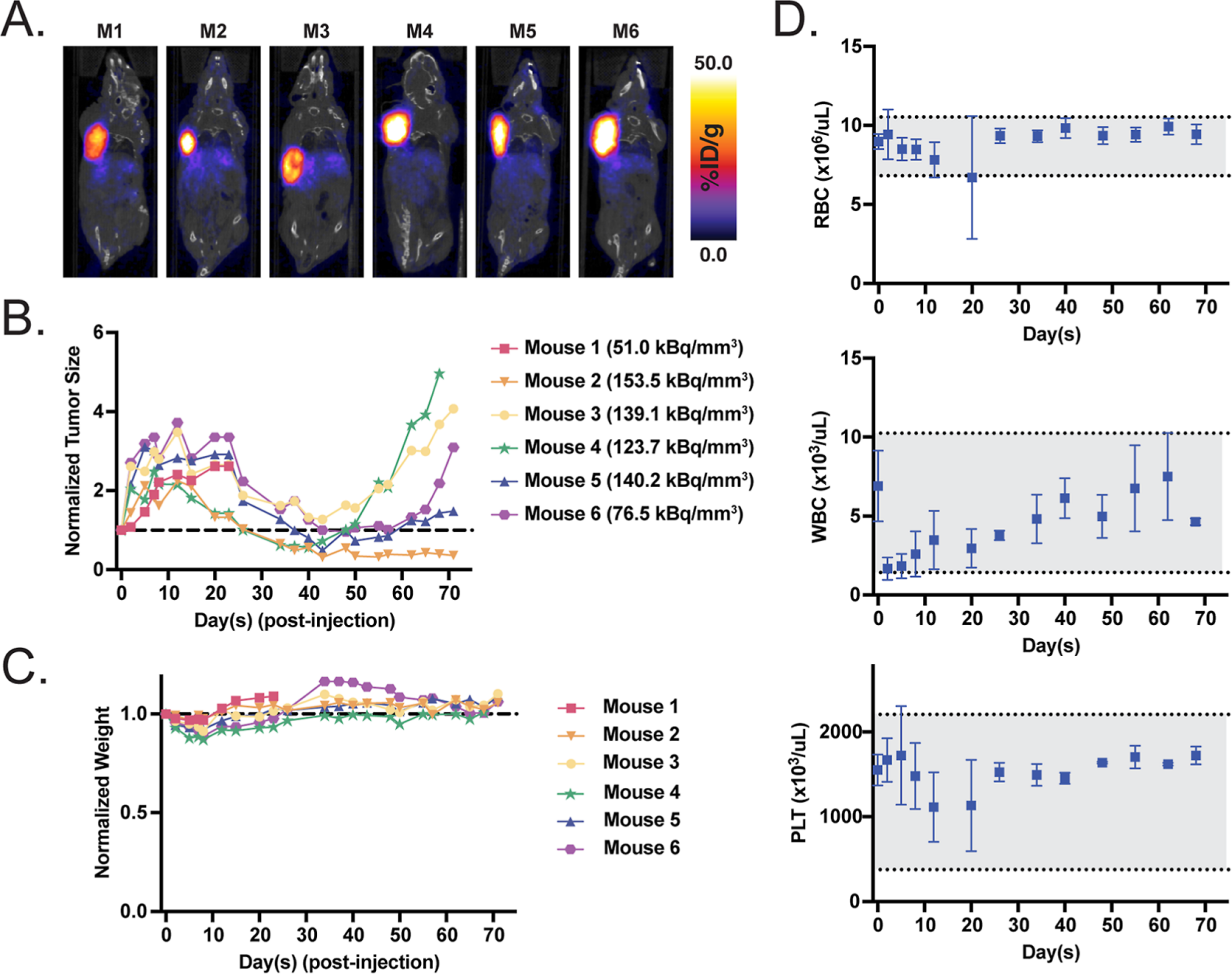
(A) Coronal PET images of the six mice from the longitudinal therapy study acquired 48 h after the administration of [^89^Zr]­Zr-DFO-[^177^Lu]­Lu-DOTA-^TzAz^A33 (100 μCi ^89^Zr, 200 μCi ^177^Lu, 33.3 μg, in 100 μL 0.9% sterile saline); (B) normalized tumor size as a function of time after the administration of the radioimmunoconjugate; (C) normalized body weight as a function of time for each mouse; (D) hematological parameters for each of the mice in the study. RBC: red blood cell count (reference range: 6.8–10.5 × 10^6^/μL); WBC: white blood cell count (reference range: 1.4–10.3 × 10^3^/μL); PLT: platelet count (reference range: 376–2206 × 10^3^/μL).

During the experiment, the xenografts experienced an initial period of growth followed by (in most cases) contraction in response to radioimmunotherapy. Despite receiving a constant amount of [^89^Zr]­Zr-DFO-[^177^Lu]­Lu-DOTA-^TzAz^A33, the PET-derived ^177^Lu activity concentrations in the tumor tissue spanned a significant range: ∼50 to ∼150 kBq/mm^3^. Interestingly, the mouse that had to be euthanized very early in the experiment (i.e., day 23) exhibited the lowest tumoral activity concentration (i.e., 51 kBq/mm^3^), while the two mice that responded best to radioimmunotherapy displayed the highest tumoral activity concentrations (i.e., 153 and 140 kBq/mm^3^) (Figure 6B and Table S19). It is important to note, however, PET-derived dosimetry data did not perfectly predict response to therapy, as no clear relationships between activity concentration and response were observed in the mice between these two extremes. This lack of a clear overarching pattern may stem from the relatively small sample size of this pilot study (i.e., *n* = 6) or from differences in the volume and vascularization of the xenografts themselves. Nonetheless, these data suggest that the ^89^Zr-immunoPET-derived dosimetry data could be used to predict tumoral dosimetry—and thus, by extension, response to therapy—on an individualized basis. Finally, the dual-labeled probe was well tolerated. No significant deviations in weight or red blood cell count were observed; white blood cell and platelet counts dropped steeply immediately after injection but remained within a normal range and rose over time (Figure 6C,D).

## Conclusion

In this study, we report the development and comprehensive in vitro and in vivo evaluation of a dual-labeled radioimmunoconjugate for simultaneous immunoPET and radioimmunotherapy. By harnessing a trio of orthogonal selective chemistries (i.e., the SPAAC, IEDDA, and cysteine-PODS ligations) we have created a versatile scaffold—i.e., TzAz-PODS—that supports both site-selective bioconjugation and the attachment of two different cargoes. The ^89^Zr/^177^Lu-labeled radioimmunoconjugate at the center of this work was shown to be both highly stable and immunoreactive and exhibited excellent in vivo performance in a murine model of colorectal carcinoma. Critically, the ^89^Zr-PET images acquired with the reagent were shown to accurately reflect the ^177^Lu dosimetry of the probe and, potentially, predict response to therapy.

The ^89^Zr/^177^Lu-labeled radioimmunoconjugate at the heart of this work clearly demonstrates the benefits of this single-agent approach to radiotheranostics. The dual-labeled probe produced excellent in vivo performance with a pharmacokinetic profile identical to an analogous ^89^Zr-only labeled antibody. Even more importantly, the PET images derived from the radioimmunoconjugate’s ^89^Zr label effectively predicted the probe’s ^177^Lu dosimetry and thus could offer insight into the response of patients to targeted radiotherapy. The initial in vivo experiments and subsequent longitudinal radioimmunotherapy study served to illustrate another important facet of the system: the ratio of ^89^Zr:^177^Lu activities can be easily altered (1:1 in the former and 1:2 in the latter). This may prove especially valuable in the future in light of the advent of total-body PET, as very small activities of ^89^Zr could be used to inform upon radioimmunotherapy with much higher activities of ^177^Lu.^36–38^ Finally—though no less importantly—TzAz-PODS itself represents a highly promising tool even outside of radiopharmaceutical chemistry, as the trifunctional reagent could be used to facilitate the site-selective modification of myriad biomolecules with two different cargoes in a stable, well-defined, and homogeneous manner.

Despite these advantages, it is important to note that this technology is not without its limitations. Perhaps the most obvious is its technical complexity, as the platform requires several different reactions to produce the final product: (i) bioconjugation; (ii) click of the first chelator; (iii) radiolabeling; (iv) labeling of second chelator; and (v) second chelator attachment. This could prove challenging in the context of clinical production, though the logistical and regulatory benefit of having only a single agent may outweigh these challenges. Second, the use of a dual-labeled agent for simultaneous PET and radioimmunotherapy inherently precludes the use of the agent for pretreatment “scout” scans that could help identify patients that are likely to respond to therapy. That said, it would always be possible to perform theranostic imaging with the singly labeled variant of the drug—i.e., [^89^Zr]­Zr-DFO-DOTA-^TzAz^A33—to identify potential responders prior to simultaneous imaging and therapy with the dual-labeled analogue.

In the end, this investigation has yielded both a versatile scaffold for the development of multifunctional biomolecular tools—TzAz-PODS—as well as an innovative application of this technology: a ^89^Zr- and ^177^Lu-labeled radioimmunoconjugate for simultaneous immunoPET and radionuclide therapy. We are currently working to streamline radiochemical processes for this strategy, deploy this bioconjugation platform with other cargoes, develop dual-labeled peptide- and small molecule-based radiotheranostics, and explore synergies offered by radiopharmaceuticals labeled with radionuclidic pairs beyond ^89^Zr and ^177^Lu.

## Supplementary Material



## Notes

Reagents and general procedures; detailed experimental methods; instrument calibration curves; gel electrophoresis results; cellular immunofluorescence images; immunoreactivity data; stability data; SE-HPLC and radio-iTLC chromatograms; ELISA results; PET/CT images; tables and graphs of biodistribution data; ^1^H- and ^13^C NMR spectra; dosimetry data; and addition reaction schemes (PDF)

## Abbreviations

Calcd: calculated
CT: computed tomography
DCM: dichloromethane
DIPEA: *N*,*N*-diisopropylethylamine
DFO: desferrioxamine
DMF: dimethylformamide
DOL: degree of labeling
DOPTI: 1,4,7,10-tetraazacyclododecane-1,4,7,10-tetrakis­(methylenephosphonic acid)
DOTA: 1,4,7,10-tetraazacyclododecane-1,4,7,10-tetraacetic acid
ESI: electrospray ionization
EtOAC: ethyl acetate
EtOH: ethanol
HATU: 1-[bis­(dimethylamino)­methylene]-1*H*-1,2,3-triazolo­[4,5-*b*]­pyridinium 3-oxide hexafluorophosphate
HPLC: high performance liquid chromatography
HR-MS: high resolution mass spectrometry
%ID/g: percent injected dose per gram
iTLC: instant thin-layer chromatography
LR-MS: low resolution mass spectrometry
MeCN: acetonitrile
MeOH: methanol
MHz: megahertz
PBS: phosphate-buffered saline
PBS-T: phosphate-buffered saline with tween-20
PET: positron emission tomography
PSMA: prostate-specific membrane antigen
*R*_t_: retention time
SDS-PAGE: sodium dodecyl sulfate polyacrylamide gel electrophoresis
TATE: Tyr3-octreotate
THF: tetrahydrofuran
TRAP: 1,4,7-triazacyclononane-1,4,7-tris­(methylenephosphinic acid)
TLC: thin layer chromatography
UV: ultraviolet
