# Supplementary material for: Selective Chemistry Enables Simultaneous ImmunoPET and Radioimmunotherapy with a Dual-Labeled Probe
Source: JACS Au. 2026 Jun 25;6(7):4028–38. doi: 10.1021/jacsau.6c00555 (PMC13417245; doi:10.1021/jacsau.6c00555)
Supplement: Supplementary file 1 [file au6c00555_si_001.pdf]

Supporting Information

Selective Chemistry Enables Simultaneous ImmunoPET and Radioimmunotherapy with a Dual-Labeled Probe

Wei-Siang Mark Kao[1,2,*], Camilla Grimaldi[1,2,3,*], Zachary V. Samuels[1,2,4], Gina Dehlavi[1,2,3], Emilia Strugala[1], Mike Cornejo[1,2,4], Joni Sebastiano[1,2,3], Lukas M. Carter[5] and Brian M. Zeglis[1,2,3,4,6,δ]

[1]Department of Chemistry, Hunter College, The City University of New York, New York, NY, USA, 10065

[2]Department of Radiology, Memorial Sloan Kettering Cancer Center, New York, NY, USA 10065

[3]Ph.D. Program in Biochemistry, Graduate Center of City University of New York, New York, NY, USA 10065

[4]Ph.D. Program in Chemistry, Graduate Center of City University of New York, New York, NY, USA 10065

[5]Department of Medical Physics, Memorial Sloan Kettering Cancer Center, New York, NY, USA 10065

[6]Department of Radiology, Weill Cornell Medical College, New York, NY, USA 10065

*These authors contributed equally to this work

Table of Contents

List of Abbreviations 3

Supplementary Materials and Methods 4

Supplementary Figures and Figure Captions..13

Supplementary Tables and Table Legends..39

Supplementary Schemes 51

List of Abbreviations

Calcd	Calculated
CT	Computed Tomography
DCM	Dichloromethane
DFO	Desferrioxamine
DIPEA	*N,N*-diisopropylethylamine
DMF	Dimethylformamide
DOL	Degree of labeling
DOTA	1,4,7,10-tetraazacyclododecane-1,4,7,10-tetraacetic acid
ESI	Electrospray ionization
EtOAc	Ethyl acetate
EtOH	Ethanol
HATU	1-[Bis(dimethylamino)methylene]-1H-1,2,3-triazolo[4,5-b]pyridinium 3-oxide hexafluorophosphate
HPLC	High performance liquid chromatography
HRMS	High resolution mass spectrometry
%ID/g	Percent injected dose per gram
ITLC	Instant Thin-Layer Chromatography
LC	Liquid Chromatography
LRMS	Low resolution mass spectrometry
MeCN	Acetonitrile
MeOH	Methanol
MHz	Megahertz
PBS	Phosphate-buffered saline
PBS-T	Phosphate-buffered saline with Tween-20
PET	Positron Emission Tomography
R_t	Retention time
SDS-PAGE	Sodium Dodecyl Sulfate Polyacrylamide Gel Electrophoresis
THF	Tetrahydrofuran
TLC	Thin layer chromatography
UV	Ultraviolet

Supplementary Materials and Methods

All reagents and materials were purchased from Sigma-Aldrich and Fisher Scientific and used as received without further purification unless otherwise noted. All water used was ultra-pure (>18.2 MΩcm^{-1}); hexanes, ethyl acetate (EtOAc), dichloromethane (DCM), diethyl ether (Et_2O), dimethyl sulfoxide (DMSO), and dimethylformamide (DMF) were acquired from Sigma-Aldrich in molecular biology grade (>99.9%). Phosphate-buffered saline (PBS) and filtered Chelex-PBS were prepared using standard protocols. IRDye® 800CW-DBCO, SulfoCy5-TCO, and BCN-AlexaFluor488 were purchased from Li-CorBio™ (Lincoln, NE, USA), BroadPharm (San Diego, CA, USA), and Lumiprobe (Westminster, MD, USA), respectively. ^{89}Zr was produced and purified at Memorial Sloan Kettering Cancer Center (MSKCC), and ^{177}Lu was purchased from ITM Radiopharma (ITM Isotope Technologies, Munich, Germany). Deferoxamine mesylate salt and *p*-SCN-Bn-DOTA (B-205) were purchased from Macrocyclics (Plano, TX, USA).

Organic reactions were monitored by thin-layer chromatography on TLC aluminum sheets (Millipore Sigma TLC aluminum sheet, Silica gel 60 F_{254}) or by LC/MS (Waters UPLC-MS system, direct-injection, C_4, C_8, or C_{18} column mode, 1–10 µL, ESI). TLC plates were visualized by hand-held UV illumination (UVGL-15, 254/365 nm, 4W) or developed with potassium permanganate $KMnO_4$ stain solution (Cat#: P1483, TCI, Tokyo, Japan). Flash chromatography was carried out using a Biotage® Isolera™ system (Isolera™ One, Biotage, Uppsala, Sweden) with Sfär silica, Sfär KP-Amino, or Sfär C_{18} Duo columns. HPLC was performed using Shimadzu HPLC equipped with a Jupiter reversed-phase C_{18} column (4.6 × 250 mm, 5 µm) or a Cytiva Superdex™ 200 small-scale size-exclusion column (10 × 300 mm, 10/300 GL).

NMR spectra (^{1}H and ^{13}C) were obtained using a 600 MHz or 500 MHz Bruker spectrometer and analyzed using Mestrenova 14.3. ^{1}H and ^{13}C chemical shifts (δ) were referenced to residual solvent peaks. The following residual solvent peaks were chosen for ^{1}H NMR: $CDCl_3$ (7.26 ppm), CD_3OD (3.31 ppm), and $(CD_3)_2SO$ (2.50 ppm). The following residual solvent peaks were chosen for ^{13}C NMR: $CDCl_3$ (77.16 ppm), CD_3OD (49.00 ppm), and $(CD_3)_2SO$ (39.52 ppm). The following abbreviations were used to define ^{1}H NMR peaks: s, singlet; d, doublet; t, triplet; q, quartet; m, multiplet. Low-resolution electrospray ionization (ESI) and High-resolution electrospray ionization (ESI) mass spectra were obtained at the Memorial Sloan Kettering Cancer Center (MSKCC) Nuclear Magnetic Resonance Analytical Core Facility (NMRACF). MALDI mass spectrometry was conducted at the Alberta Proteomics and Mass Spectrometry Facility (University of Alberta; Edmonton, AB, Canada).

Instrumentation

All instruments were maintained and calibrated in compliance with established quality control standards and procedures. UV-Vis measurements were taken by Thermo Scientific NanoDrop One C UV-

Vis spectrophotometer (ThermoFisher Scientific, Waltham, MA, USA). The bioimages for immunocytochemistry were obtained by using a Nikon A1 confocal microscope (Nikon; Tokyo, Japan). Radioactivity measurements were performed by using CRC-15R Dose Calibrator (Capintec; Ramsey, NJ) and an automatic Wizard2 gamma counter (PerkinElmer, Waltham, MA). Radio-TLC was performed using a Bioscan AR-2000 imaging scanner on iTLC-SG glass microfiber chromatography paper (SGI001, Agilent Technologies).

Synthesis of Az-CN

Az-CN was prepared via previously published protocols.[1] In brief, to 4-bromobutyronitrile (0.4 g, 2.70 mmol, 1.0 eq.) in DMSO (6.0 mL) was added sodium azide (0.23 g, 3.51 mmol, 1.3 eq.). The mixture was stirred at 80 °C for 16 h. The crude product was extracted using diethyl ether and water, and the organic layer was collected, washed with brine, dried over anhydrous Na_2SO_3, and concentrated *in vacuo*. The product was collected without further purification as a light-yellow oil (0.25 g; Mw = 110.12 g; yield 83%). ^{1}H NMR (500 MHz, DMSO-d_6): δ = 3.38 (t, *J* = 5.0 Hz, 2H), 2.38 (t, *J* = 5.0 Hz, 2H), 1.83 (quintet, *J* = 5.0, 10.0 Hz, 2H). LRMS (ESI) m/z: Calcd. for $C_4H_7N_4^+$ $[M+H]^+$: 111.1; Found: 111.1.

Synthesis of TzAz-COOH

4-cyanophenylacetic acid (41 mg, 0.25 mmol, 1.0 eq.), Az-CN (0.22 g, 1.98 mmol, 7.8 eq.), and 3-mercaptopropionic acid (27.1 mg, 0.26 mmol, 1.02 eq.) were added to a round-bottom flask under N_2 and cooled to 0 °C using ice bath. Hydrazine hydrate (128.2 mg, 4 mmol, 16.0 eq.) was then added dropwise to this mixture at 0 °C. The mixture was then stirred at 40 °C for 16 h under N_2, and the reaction was monitored by TLC. After the reaction had completed, it was poured into water gently and cooled down to 0 °C. Sodium nitrite (0.28 g, 4 mmol) in water (1.0 mL) was added to the mixture and stirred for 10 minutes, followed by the dropwise addition of 1 M HCl until the pH of the mixture reached 2.0-3.0. The mixture was extracted with EtOAc and water and then washed with brine. The combined organic layers were dried over anhydrous Na_2SO_4, concentrated *in vacuo*, and purified via Biotage Isolera One automated flash column chromatography with a silica gel column (EtOAc:MeOH = 30:1) to obtain TzAz-COOH as a pink solid (60.6 mg, Mw = 299.29, yield 81%).^{1}H NMR (500 MHz, CD_3OD-d_4): δ = 8.53 (d, *J* = 5.0 Hz, 2H), 7.57 (d, *J* = 5.0 Hz, 2H), 3.75 (s, 2H), 3.54 (t, *J* = 5.0 Hz, 2H), 3.45 (t, *J* = 5.0 Hz, 2H), 2.28 (m, 2H). ^{13}C NMR (150 MHz, CD_3OD-d_4): δ = 175.07, 170.67, 165.51, 141.37, 132.10, 131.47, 128.93, 51.79, 42.10, 32.91, 28.10. LRMS (ESI) m/z: Calcd for $C_{13}H_{14}N_7O_2^+$ $[M+H]^+$: 300.1; Found: 300.1.

Synthesis of TzAz-PODS

To TzAz-COOH (5.7 mg, 0.02 mmol, 1.05 eq.) in DMF (1.0 mL) was added HATU (8.2 mg, 0.02 mmol, 1.2 eq.) and DIPEA (3.48 µL, 0.02 mmol, 1.2 eq.). The mixture was allowed to stir at 0 °C for 10 minutes, and PODS (10.0 mg, 0.018 mmol, 1.0 eq.) was slowly added into the solution for an additional 2 hours. The crude product was purified via Biotage Isolera One automated flash column chromatography on a C_{18} column using 10% of $MeCN/H_2O$ (v/v) over 20 minutes to afford the product as pink solid (14.2 mg, Mw = 822.89, yield 86%). ^1H NMR (500 MHz, $CDCl_3$): δ = 9.86 (bs, 1H), 8.53 (d, *J* = 5.0 Hz, 2H), 8.03 (d, *J* = 5.0 Hz, 2H), 7.76 (d, *J* = 10.0 Hz, 2H), 7.53 (d, *J* = 10.0 Hz, 2H), 6.79 (bs, 1H), 3.67–3.35 (m, 16H), 2.68–2.60 (m, 3H), 2.32 (dd, *J* = 10.0, 15.0 Hz, 2H), 1.77–1.59 (m, 12H). ^{13}C NMR (150 MHz, $CDCl_3$): δ = 164.10, 161.73, 140.30, 130.29, 128.82, 128.39, 119.65, 70.49, 70.41, 69.96, 69.78, 50.64, 43.70, 43.02, 38.74, 38.10, 31.45, 31.99, 29.05, 28.49, 27.06. HRMS (ESI) m/z: Calcd for $C_{12}H_{46}N_{12}O_9NaS^+$ $[M+Na]^+$: 845.3124; Found: 845.3126.

Synthesis of TCO-DFO

To TCO-NHS (10.0 mg, 0.037 mmol, 1.0 eq.) in THF (2.0 mL) was added deferoxamine mesylate (24.0 mg, 0.037 mmol, 1.0 eq.) at 0 °C in an ice bath. DIPEA (7.70 µL, 0.056 mmol, 1.5 eq.) was slowly added to the mixture, and the mixture was allowed to stir at room temperature for 16 h. After the reaction had completed, the DCM was removed via rotary evaporators. The crude product was purified by Biotage Isolera One automated flash column chromatography with a C_{18} column using 10% of $MeCN/H_2O$ (v/v) over 15 minutes to afford colorless oil (24.3 mg, Mw = 712.88, yield 92%). ^1H NMR (500 MHz, CD_3OD-d_4): δ = 8.52 (bs, 1H), 5.60 (m, 1H), 5.48 (m, 1H), 4.52 (s, 3H), 3.61 (m, 3H), 3.18 (m, 2H), 3.07 (t, *J* = 10.0 Hz, 2H), 2.77 (t, *J* = 10.0 Hz, 2H), 2.47 (t, *J* = 10.0 Hz, 2H), 2.33 (m, 2H), 2.09 (s, 3H), 1.93 (m, 3H), 1.64–1.55 (m, 5H), 1.51–1.45 (m, 4H), 1.35–1.20 (m, 4H). ^{13}C NMR (150 MHz, CD_3OD-d_4): δ = 171.97, 171.30, 170.13, 155.75, 134.91, 132.52, 78.85, 47.08, 46.77, 33.75, 32.15, 30.58, 29.89, 29.13, 28.81, 27.55, 26.01, 23.48, 23.34, 20.34. LRMS (ESI) m/z: Calcd for $C_{34}H_{61}N_6O_{10}^+$ $[M+H]^+$: 713.8; Found: 713.8.

Synthesis of BCN-DOTA

To a solution of endo-BCN-PEG2-amine (5.0 mg, 0.015 mmol, 1.0 eq.) in DMSO (200 µL) was added DIPEA (26 µL, 0.15 mmol, 10.0 eq.), and the resultant solution was stirred for 5 minutes at room temperature. To this mixture was added *p*-SCN-Bn-DOTA (11.4 mg, 0.017 mmol, 1.1 eq.), and the reaction was stirred at room temperature overnight. The crude product was purified by Biotage Isolera One automated flash column chromatography with a C_{18} column (gradient $MeCN/H_2O$ (v/v) + 0.1%TFA, 0% MeCN to 100% in 35 minutes) to afford colorless oil (12.1 mg, Mw = 876.04, yield, 92%). ^1H NMR (500 MHz, DMSO-d_6): δ = 9.59 (bs, 1H), 7.68 (bs, 1H), 7.45 (d, *J* = 10.0 Hz, 2H), 7.23 (bs, 2H), 7.09 (d, *J* =

10.0 Hz, 2H), 4.04 (d, J = 10.0 Hz, 2H), 3.58–3.50 (m, 10H), 3.15–2.90 (m, 10H), 2.64 (s, 2H), 2.26 (s, 2H), 2.23–2.10 (m, 6H), 1.53–1.51 (m, 2H), 1.28–1.26 (m, 2H), 1.25 (s, 2H), 0.90–0.83 (m, 2H). HRMS (ESI) m/z: Calcd for $C_{41}H_{62}N_7O_{12}S^+$ $[M+H]^+$: 876.4177; Found: 876.4194.

Synthesis of ^{TzAz}5B1 and ^{TzAz}A33

To 5B1 or A33 (11.4 mg/mL in PBS, 100 μL) in PBS (1029 μL) was added TCEP (4 mg/mL in H_2O, 1.5 μL, 20,0 eq.). The pH of this solution was adjusted to 7.4 with 0.1 M $Na_2CO_{3(aq)}$, and the mixture was incubated at 37 °C and 500 rpm for 30 minutes. Subsequently, TzAz-PODS (24.0 mg/mL in DMSO, 1.4 μL, 20.0 eq.) was added gradually, and the mixture was incubated at 37 °C and 500 rpm for 30 minutes. The resulting immunoconjugates — ^{TzAz}5B1 and ^{TzAz}A33 — were purified via size-exclusion chromatography using PD-10 columns (GE Healthcare; Chicago, IL, USA) and concentrated with 2 mL Amicon Ultra centrifugal filters with a 50 kDa molecular weight cutoff (MWCO; MilliporeSigma).

General Procedure for Click Chemistry

To ^{TzAz}A33 or ^{TzAz}5B1 (100 μL of a 15 mg/mL solution in PBS) in PBS (890 μL) was added TCO-DFO or TCO-Cy5 (10.0 mg/mL, 10.0–20.0 eq.). The mixture was incubated at 37 °C and 500 rpm for 30 minutes, followed by the gradual addition of second click reagent: BCN-DOTA, BCN-AF488, DBCO-IR800 (10.0 mg/mL, 20.0 eq.). This mixture was then incubated under the same conditions for another 30 minutes. The resulting immunoconjugates were purified via size-exclusion chromatography using PD-10 columns (GE Healthcare; Chicago, IL, USA) and concentrated with 2 mL Amicon Ultra centrifugal filters with a 50 kDa molecular weight cutoff (MWCO; MilliporeSigma).

SDS-PAGE Electrophoresis Analysis

SDS-PAGE was used to verify the purity of each immunoconjugate as well as the site-selectivity of the modifications. The samples were prepared by adding 5 μg of each immunoconjugate with LDS loading buffer (12.5 μL) and MilliQ H_2O to a total volume of 50 μL. All samples were loaded onto a NuPAGE™ 4–12% Bis-Tris gel and run in MOPS SDS Running buffer. 5 μL of Novex Sharp Pre-Stained Protein Ladder was loaded to the outermost wells as the size reference. The gel was run at 80 V for about 180 minutes until the lowest molecular weight ladder band reached the bottom of the gel. Then, the gel was washed with MilliQ H_2O three times. The fluorescence image was acquired using Typhoon FLA 7000 instrument and analyzed with Typhoon FLA 7000 Control Software. After the fluorescence imaging, the gel was further stained with SimplyBlue™ SafeStain (Thermofisher) for an hour and washed with MilliQ H_2O three times. The gel was imaged using a LI-COR Odyssey® CLx instrument and analyzed using Image Studio™ Acquisition Software.

Radiolabeling with Zirconium-89

The DFO-bearing immunoconjugates were radiolabeled with $[^{89}Zr]Zr^{4+}$ following the standard published protocols.[2,3] Briefly, to a solution of $[^{89}Zr]$Zr-oxalate was added ten times volume of HEPES buffer to adjust the pH of the solution to 7.0. After $[^{89}Zr]$Zr-oxalate was neutralized, the $[^{89}Zr]Zr^{4+}$ solution was added to a 1 mg/mL solution of each of the immunoconjugates at a specific activity of 3–4 mCi/mg. The mixture was then allowed to incubate at 37 $^{\circ}$C and 500 rpm for 15 minutes. The crude mixture was purified using PD-10 columns to remove excess unlabeled $[^{89}Zr]Zr^{4+}$ and concentrated with 2 mL Amicon Ultra centrifugal filters with a 50 kDa molecular weight cutoff (MWCO; MiliporeSigma). The radiolabeling reactions and radioimmunoconjugates were monitored via radio-iTLC and size-exclusion column HPLC to ensure the products meet quality control standard for further *in vitro* and *in vivo* studies.

Radiolabeling with Lutetium-177

$[^{89}Zr]$Zr-DFO-^{TzAz}A33 was radiolabeled with $[^{177}Lu]Lu^{+3}$ via the strain-promoted azide–alkyne ligation with $[^{177}Lu]$Lu-DOTA-BCN. To this end, BCN-DOTA (10 mg/mL, 5.0 µL) in NaOAc (0.4 M, pH 5.0, 195 µL) was added to $[^{177}Lu]Lu^{+3}$ at a specific activity of 10-15 mCi/mg, and the mixture was incubated at 95 $^{\circ}$C and 500 rpm for 10–15 minutes. The reaction was cooled to RT, and the $[^{177}Lu]$BCN-Lu-DOTA was added (without further purification) to a solution of $[^{89}Zr]$Zr-DFO-^{TzAz}A33 in Chelex-treated PBS. The mixture was incubated at 37 $^{\circ}$C and 500 rpm for 30 minutes. The crude mixture was then purified using a PD-10 column to remove excess unlabeled $[^{177}Lu]Lu^{3+}$ and concentrated with 2 mL Amicon Ultra centrifugal filters with a 50 kDa molecular weight cutoff (MWCO; MiliporeSigma).

Cell Culture of BxPC-3

The human pancreas adenocarcinoma cell line BxPC-3 (CRL-1687) was purchased from American Type Culture Collection (ATCC) and was cultured and maintained using Roswell Park Memorial Institute (RPMI) Medium supplemented with 10% fetal calf serum, 10 mM HEPES, 2 mL L-glutamine, 1 mM sodium pyruvate, 1.5 g/L sodium bicarbonate, 4.5 g/L glucose, 100 units/mL penicillin, and 100 units/mL streptomycin in an incubator at 37 $^{\circ}$C and 5% $CO_{2(g)}$. The cells were harvested and passaged upon reaching 80% confluency with Gibco TrypLE Express enzyme (1×) with phenol red (Cat# 12605010, ThermoFisher Scientific, Waltham, MA, USA) for 5 minutes. The cells were also stained using cell stainers (MACS SmartStainers, 30 and 70 µm; Miltenyi Biotec; Auburn, CA, USA) to ensure a homogeneous cell suspension mixture prior to any experiments or xenografting.

Cell Culture of SW1222

The human colorectal adenocarcinoma cell line SW1222 was obtained from the Ludwig Institute of Cancer Research and was cultured and maintained using Iscove's Modified Dulbecco's Medium (IMDM) supplemented with 10% fetal calf serum, 100 units/mL penicillin, and 100 units/mL streptomycin in an incubator at 37 °C and 5% $CO_{2(g)}$. The cells were harvested and passaged upon reaching 80% confluency with Gibco TrypLE Express enzyme (1×) with phenol red (Cat# 12605010, Thermo Fisher Scientific, Waltham, MA, USA) for 6 minutes. The cells were also stained using cell stainers (MACS SmartStainers, 30 and 70 μm; Miltenyi Biotec; Auburn, CA, USA) to ensure a homogeneous cell suspension mixture prior to any experiments or xenografting.

Immunocytochemistry

Immunocytochemistry was performed and analyzed using the chamber slide system (Cat# 154917PK, Nunc™ Lab-Tek™ II CC2™ Chamber Slide System, 4 wells; ThermoFisher Scientific, Waltham, MA, USA). For cell fixation, 50,000 SW1222 or BxPC-3 cells were seeded into each chamber and left to grow for 24 hours in an incubator at 37 °C and 5% $CO_{2(g)}$ until 70–85% confluency. Then, 4% paraformaldehyde (PFA) or 10% formalin was added for 20 minutes after the cell culturing media was aspirated and removed. After the cells were fixed, the chamber was washed three times with PBS to remove PFA or formalin residue in the chamber.

ELISA Analysis of Immunoconjugates

The immunoreactivity of A33-based immunoconjugates was assessed by ELISA using recombinant human A33 antigen (Cat# 11277-H08H, SinoBiological; Paoli, PA, USA). Sterile 96-well microplates (Cat# 07-200-91, Corning™ Coster™ 96-Well Microplate; Fisher Scientific, Waltham, MA, USA) were coated with recombinant A33 antigen (1 μg per well) diluted in PBS buffer (pH 7.4) and incubated overnight at 4 °C. The wells were then washed with PBS-T three times (200 μL/well each time) and blocked with 10% BSA in PBS (200 μL) for an hour at RT. Serial dilutions of modified or unmodified A33 antibody were prepared in blocking buffer and added to the wells. After incubation for 2 hours at room temperature, the plate was washed thoroughly with PBS-T three times (200 μL/well each time). Bound antibody was detected using HRP-conjugated anti-human IgG (Cat# 62-8420, Goat anti-human IgG, HRP; ThermoFisher Scientific, Waltham, MA, USA) as the secondary antibody. Following incubation for an hour at RT and three washes with PBS-T, TMB substrate (Cat# PI34022, Thermo Scientific™ 1-Step™ TMB ELISA Substrate Solutions; ThermoFisher Scientific, Waltham, MA, USA) was added (100 μL/well), and the colorimetric reaction was stopped after 10 minutes with 1 M sulfuric acid (50 μL/well). The absorbance of each well was measured at 450 nm using a microplate reader. Binding curves were generated from

background-corrected absorbance values, and the binding profiles of each immunoconjugate were compared with that of the native A33 antibody to confirm retention of antigen–binding activity after bioconjugation and radiolabeling precursor conjugation.

Bead-Based Immunoreactivity Measurements

The bead-based radioimmunoreactivity measurements were performed according to previously published protocols.[4,5] Briefly, 60 µL of beads (10 mg/mL, Ni-NTA Magnetic Beads; ThermoFisher Scientific; Waltham, MA, USA) were washed three times with PBS (pH 7.4) containing 0.05% Tween-20 and 50mM imidazole (PBS-T). For antigen coating, test (T) and blocking (B) tubes received 20 µL of His-tag human A33 antigen (0.1 mg/mL in PBS with 1% BSA) in 380 µL PBS-T, while control (C) tubes contained 400 µL PBS-T only. All were incubated at room temperature for 30 min with rotation, followed by two washes with PBS-T. Subsequently, 400 µL PBS-T was added to all tubes; 5 µg of non-radiolabeled antibody was added to the B tubes; and all tubes received 1 µL of radioimmunoconjugate (1 µg/mL) (*i.e.* $[^{89}Zr]Zr$-DFO-^{TzAz}A33, $[^{89}Zr]Zr$-DFO-DOTA-^{TzAz}A33, and $[^{89}Zr]Zr$-DFO-$[^{177}Lu]Lu$-DOTA-^{TzAz}A33). After 30 min of incubation, the supernatants were collected, and the beads were washed twice with PBS-T, with all washes retained. Radioactivity in the beads, supernatants, and washes was quantified by gamma counting, and the immunoreactivity was calculated using the following formula:

$$\%\,Immunoreactivity = \frac{CPM_{beads}}{CPM_{beads} + CPM_{supernatants} + CPM_{washes}}$$

Stability Assays

The stability of $[^{89}Zr]Zr$-DFO-^{TzAz}A33, $[^{89}Zr]Zr$-DFO-DOTA-^{TzAz}A33, and $[^{89}Zr]Zr$-DFO-$[^{177}Lu]Lu$-DOTA-^{TzAz}A33 (1 µg/mL; n = 3) was assessed by incubation in either sterile PBS (pH 7.4) or freshly thawed human serum at 37 °C with gentle agitation. Aliquots (20–50 µL) were collected at 0, 24, 48, 72, 96, and 120 h and analyzed by radio-iTLC with an eluent of 50 mM EDTA (pH 5.0) or size-exclusion column HPLC (100% PBS, 0–35 mins).

Subcutaneous Xenografts

For *in vivo* studies, female athymic nude mice were injected with a suspension of 5×10^6 SW1222 cells in culture medium (50 µL) mixed 1:1 with Matrigel (50 µL; Corning™ Matrigel™ Matrix, Tewksbury, MA, USA) subcutaneously into the right shoulder. Tumors reached an average volume of ~100 mm^3 after approximately 12–14 days.

PET/CT Imaging

PET/CT imaging experiments were conducted via the Inveon microPET-CT (Siemens; Munich, Germany). Female athymic nude mice bearing subcutaneous SW1222 xenografts (right shoulder) were administered with the radioimmunoconjugates, [^{89}Zr]Zr-DFO-^{TzAz}A33, [^{89}Zr]Zr-DFO-DOTA-^{TzAz}A33, and [^{89}Zr]Zr-DFO-[^{177}Lu]Lu-DOTA-^{TzAz}A33, (100 μCi, 33.3 μg) in a solution of 100 μL of PBS via tail vein injection. Mice were anesthetized with the mixture of 2% isoflurane and oxygen approximately 5 min prior to PET imaging and positioned on the scanner bed. Anesthesia was maintained with 2% isoflurane throughout the procedure. Static PET scans were acquired at 4, 24, 48, 72, 120, 144, or 168 h post-injection (p.i.) depending on animal study design.

Biodistribution

All mice were administered the radioimmunoconjugates via tail vein injection as described above. At 4, 24, 48, 72, 120, 144, or 168 h post-injection (depending on experimental design), each group of mice (n = 4) were euthanized by CO_2 asphyxiation, and 16 tissues (blood, tumor, heart, lungs, liver, spleen, pancreas, stomach, small intestine, large intestine, kidneys, ovaries, bone, muscle, skin, and tail) were collected, weighed, and measured for radioactivity using a gamma counter. Tissue counts were background- and decay-corrected to the time of injection and converted to activity (μCi) using a calibration curve from known standards. The percentage of injected dose per gram (%ID/g) was calculated by normalizing each tissue's activity to the total injected dose and its mass.

Longitudinal Radioimmunotherapy Study

Radioimmunotherapy studies were conducted in female athymic nude mice bearing subcutaneous SW1222 xenografts implanted in the right shoulder. Once tumors reached approximately 400–600 mm^3, the mice (n = 6) were administered [^{89}Zr]Zr-DFO-[^{177}Lu]Lu-DOTA-^{TzAz}A33 (100 μCi ^{89}Zr + 200 μCi ^{177}Lu, 33.3 μg) in 100 μL of PBS via the tail vein. For PET imaging, mice were anesthetized with a mixture of 2% isoflurane in oxygen approximately 5 min prior to scanning and positioned on the scanner bed; anesthesia was maintained at 2% isoflurane throughout the procedure. PET scans were acquired at 48 and 72 h post-injection, according to the study design. Tumor volumes and body weights were measured twice weekly. All animals were monitored at least twice per week for signs of toxicity, including loss of appetite, body weight reduction, and skin abnormalities or infection. Defined endpoints for study termination included (1) tumor volume exceeding 2000 mm^3, (2) body weight loss greater than 10%, or (3) development of tumor necrosis.

Calibration Method for Resolving ^{89}Zr and ^{177}Lu Counts

To accurately resolve ^{89}Zr and ^{177}Lu radioactivity for dosimetry and biodistribution analysis of dual-radionuclide-labeled immunoconjugates, we developed a calibration-based method to separate the two signals using experimentally derived calibration curves. Specifically, calibration curves for ^{89}Zr and ^{177}Lu were generated by measuring gamma counts at the 208 and 909 keV energy windows for each radionuclide independently to establish the relationship between measured counts and radioactivity (*Figure S1*). This approach leverages the distinct gamma emission profiles of the two radionuclides: ^{177}Lu emits predominantly lower-energy photons (72, 113, 208, 249, and 321 keV), whereas ^{89}Zr exhibits higher-energy emissions (511 and 909 keV), with partial overlap at the 208 keV channel. Accordingly, gamma counts were first measured at 909 keV to quantify ^{89}Zr activity at time point t_1. The second measurement at 208 keV provided the combined gamma counts from both ^{177}Lu and ^{89}Zr at time point t_2.

$$A = A_0 \cdot \left(\frac{1}{2}\right)^{\frac{t}{t_{1/2}}} \quad \textit{(half-life decay equation)}$$

To resolve the individual contributions, the ^{89}Zr calibration curve at 909 keV (*Figure S1A*) was used to convert the measured 909 keV counts into ^{89}Zr activity at t_1. This activity was then used with the ^{89}Zr calibration curve at 208 keV (*Figure S1C*) to calculate the expected ^{89}Zr gamma counts at 208 keV at t_1. The background activity of ^{177}Lu at 909 keV was measured as well to ensure that there were no radioactive counts (*Figure S1B*). To account for the time difference between measurements (t_1 and t_2), radioactive decay correction based on the ^{89}Zr half-life was applied to adjust the calculated ^{89}Zr counts from t_1 to t_2 using half-life decay equation. Once the estimated ^{89}Zr contribution at 208 keV and time point t_2 was obtained, this value was subtracted from the total measured counts (^{177}Lu + ^{89}Zr) at 208 keV to yield the isolated ^{177}Lu gamma counts at t_2. These counts were subsequently converted to ^{177}Lu dosimetry using the ^{177}Lu calibration curve (*Figure S1D*). If needed, the calculated activities could be decay-corrected to other time points to meet experimental requirements. A schematic flowchart summarizing the ^{89}Zr/^{177}Lu signal resolution process is shown below.

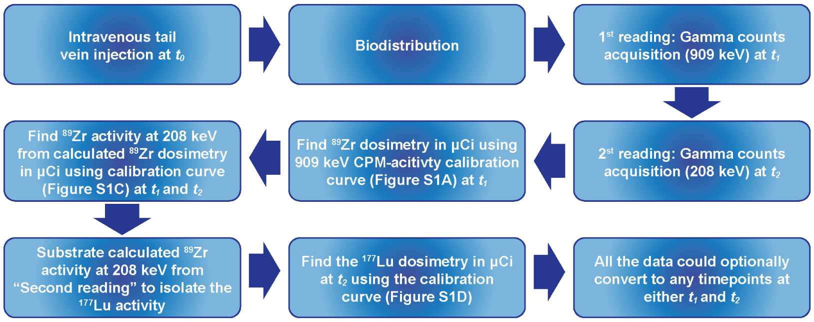

Supplemental Figures and Figure Captions

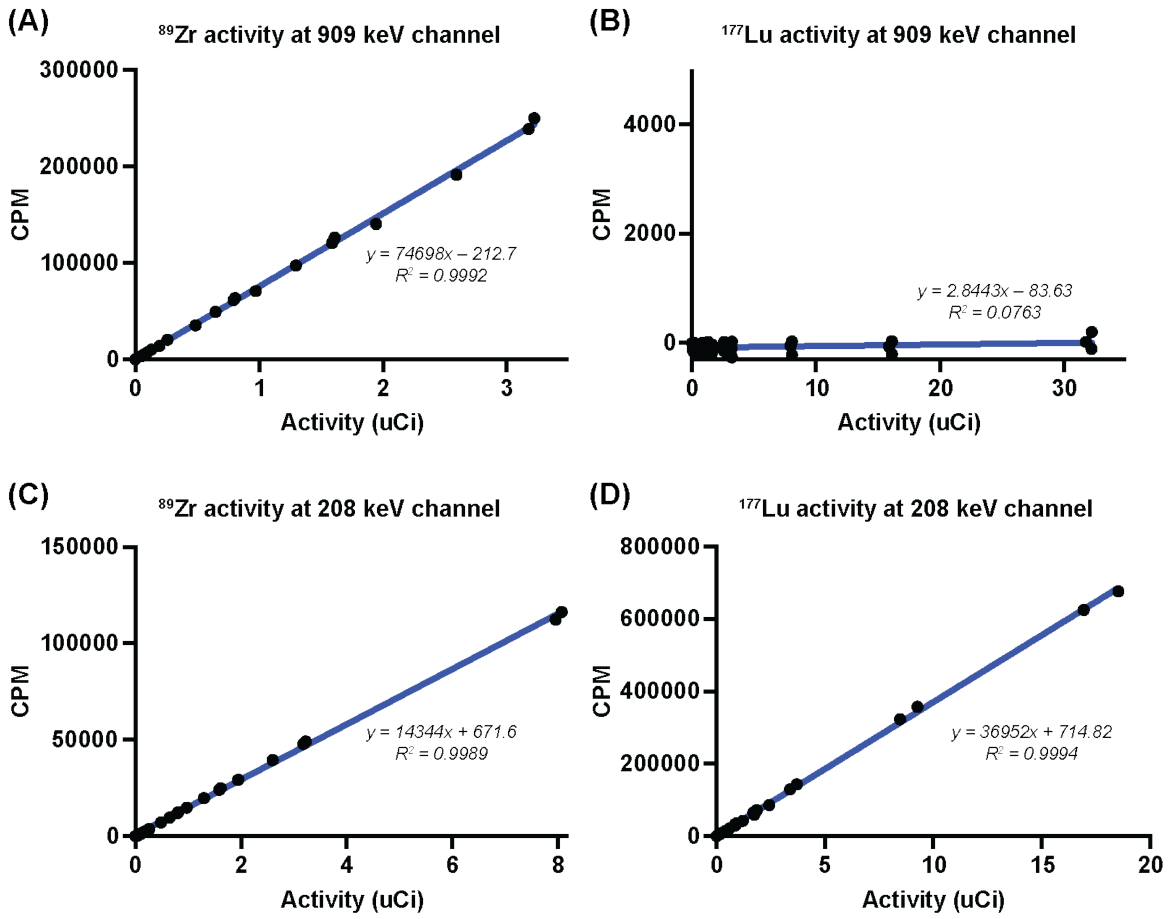

Figure S1. Calibration curves for the quantification of ^{89}Zr and ^{177}Lu using gamma spectrometry. Detector counts per minute (CPM) were measured for known activity standards of each radionuclide across their respective energy windows (909 keV for ^{89}Zr and 208 keV for ^{177}Lu). The resulting linear regression equations were used for subsequent activity calculations to resolve and quantify ^{89}Zr and ^{177}Lu contributions in experimental samples. (A) ^{89}Zr activity vs. CPM at 909 keV; (B) ^{177}Lu activity vs. CPM at 909 keV; (C) ^{89}Zr activity vs. CPM at 208 keV; (D) ^{177}Lu activity vs. CPM at 208 keV.

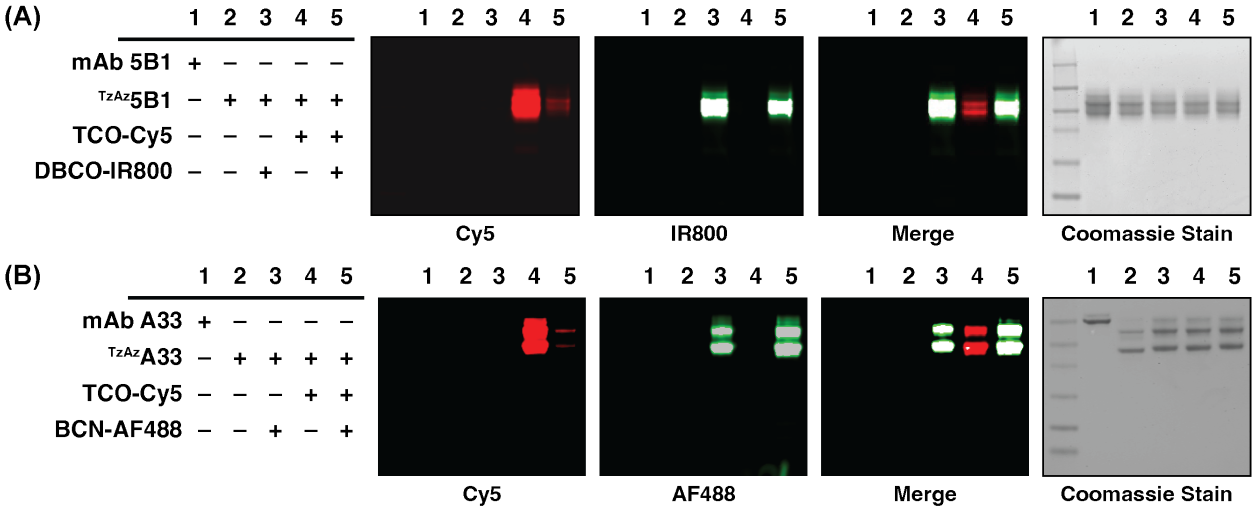

Figure S2. SDS-PAGE analysis of TzAz-PODS-modified immunoconjugates. Fluorescence images for Cy5, IR800, and Alexa Fluor 488 (AF488) channels were acquired using a Typhoon fluorescence imager prior to Coomassie Blue staining. The Coomassie-stained gel was subsequently imaged using a Li-COR Odyssey imaging system. For each sample, 2 μg of protein in a total volume of 20 μL was loaded per lane. (A) Lane 1: native mAb 5B1; Lane 2: ^{TzAz}5B1; Lane 3: IR800-^{TzAz}5B1; Lane 4: Cy5-^{TzAz}5B1; Lane 5: Cy5-IR800-^{TzAz}5B1. (B) Lane 1: native huA33; Lane 2: ^{TzAz}A33; Lane 3: AF488-^{TzAz}A33; Lane 4: Cy5-^{TzAz}A33; Lane 5: Cy5-AF488-^{TzAz}A33.

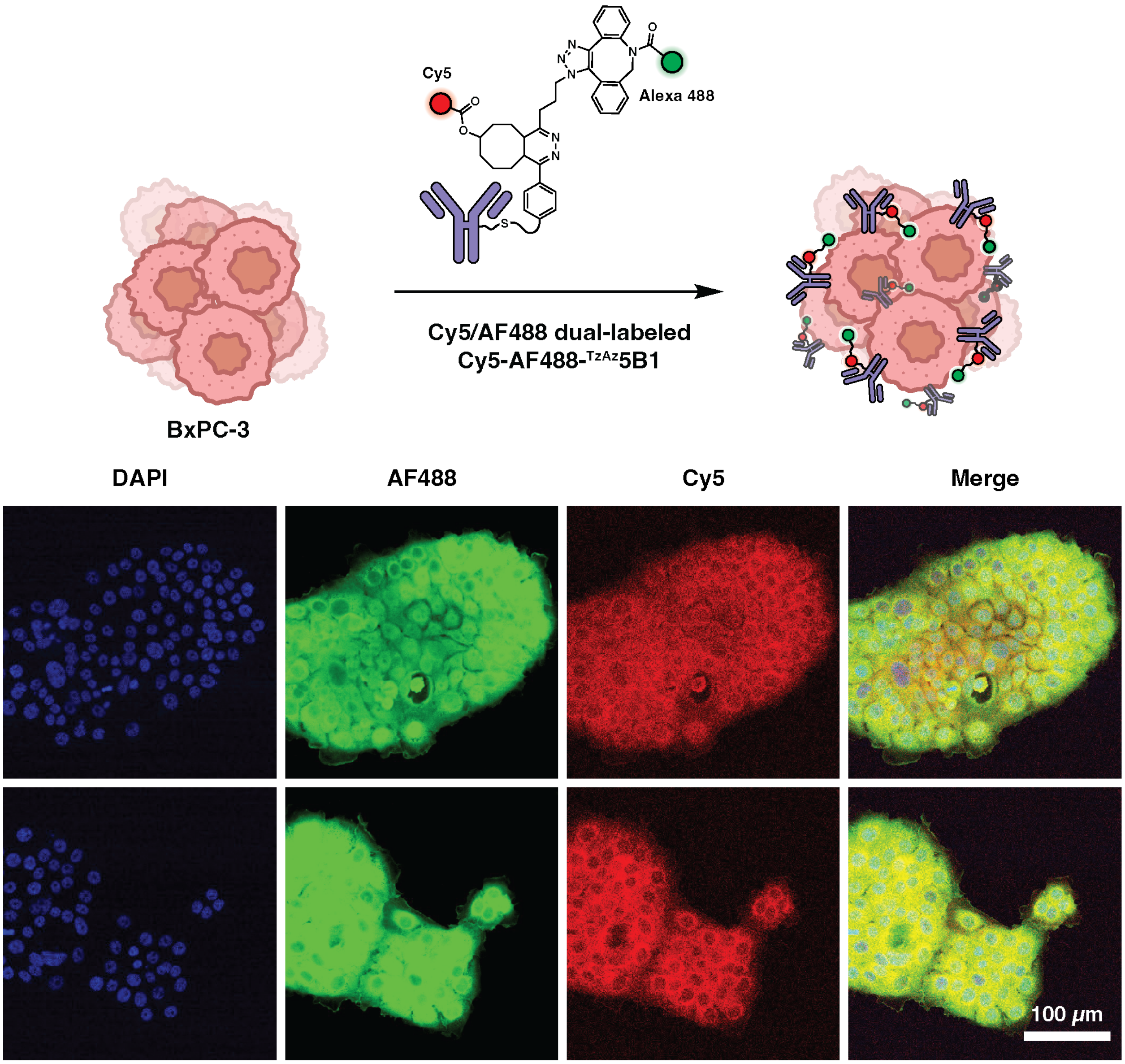

Figure S3. Confocal fluorescence images analysis of Cy5-AF488-^{TzAz}5B1 confirmed that the modified mAb 5B1 specifically targets the CA19-9 antigen expressed on BxPC-3 pancreatic ductal adenocarcinoma carcinoma cells.

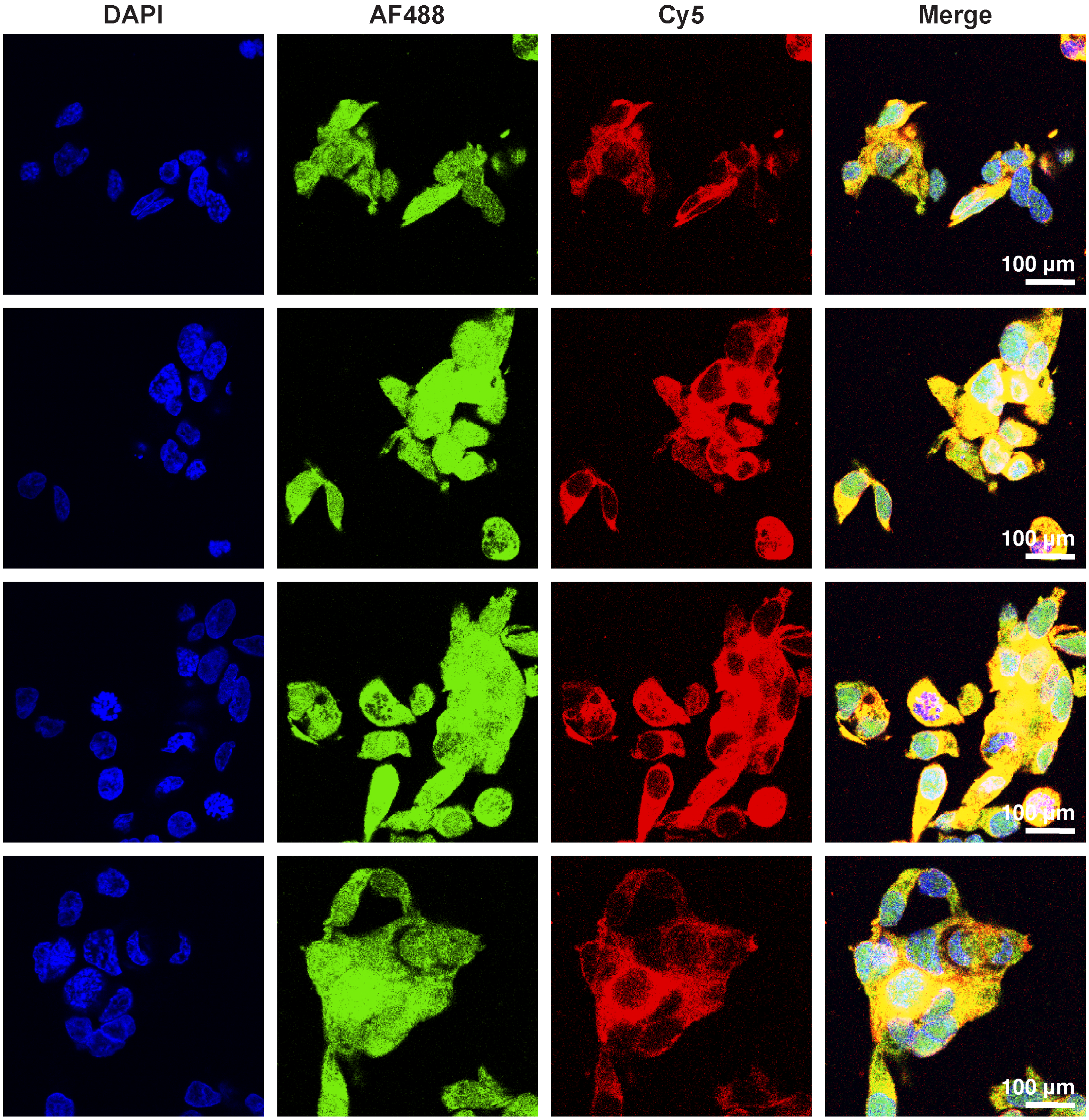

Figure S4. Confocal fluorescence images analysis of Cy5-AF488-^{TzAz}A33 confirmed that the modified mAb A33 specifically targets the A33 antigen expressed on SW1222 human colorectal cancer cells. These data correspond with that described in Figure 2C.

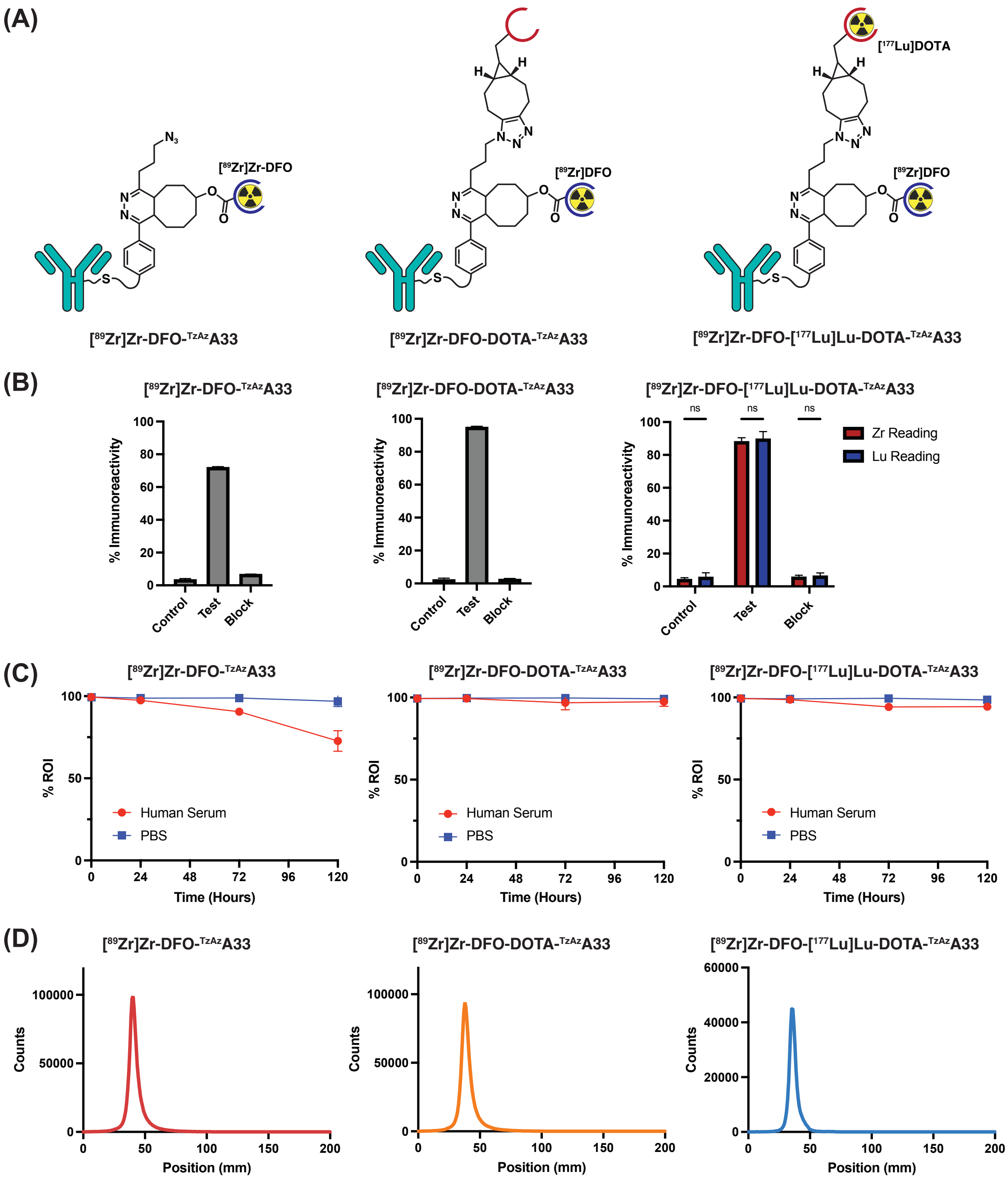

Figure S5. (A) Three radionuclide-bearing immunoconjugate constructs: [^{89}Zr]Zr-DFO-^{TzAz5}A33, [^{89}Zr]Zr-DFO-DOTA-^{TzAz}A33, and [^{89}Zr]Zr-DFO-[^{177}Lu]Lu-DOTA-^{TzAz}A33. (B) Bead-based immunoreactivity assay results of each construct. (C) 5-day stability results for each radioimmunoconjugate after incubation at 37 ^{o}C in PBS buffer (blue line) or in human serum (red line). (D) Radio-iTLC of each radioimmunoconjugate.

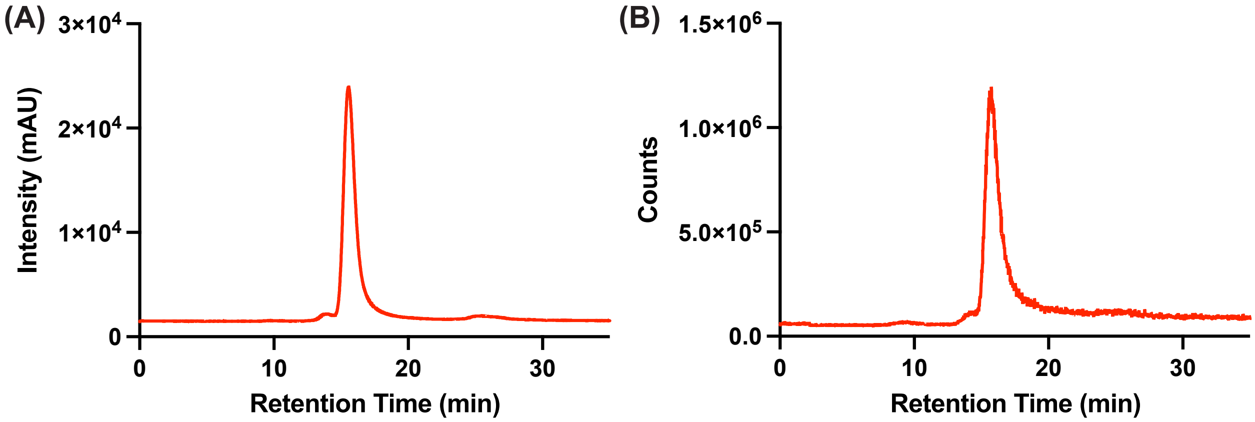

Figure S6. (A) UV-Vis (280 nm) and (B) radioactivity size exclusion radio-HPLC chromatograms of $[^{89}Zr]Zr$-DFO-^{TzAz}A33.

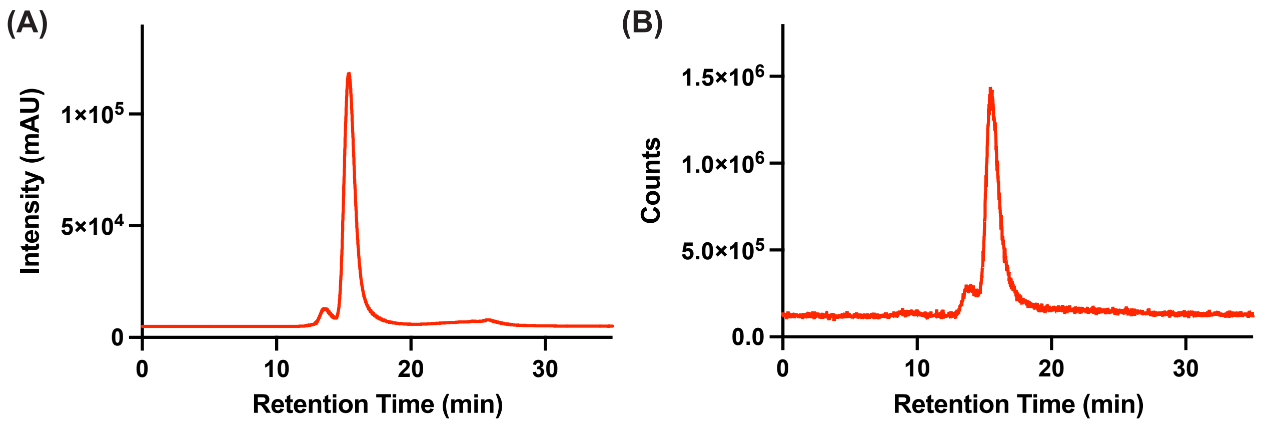

Figure S7. (A) UV-Vis (280 nm) and (B) radioactivity size exclusion radio-HPLC chromatograms of [^{89}Zr]Zr-DFO-DOTA-^{TzAz}A33.

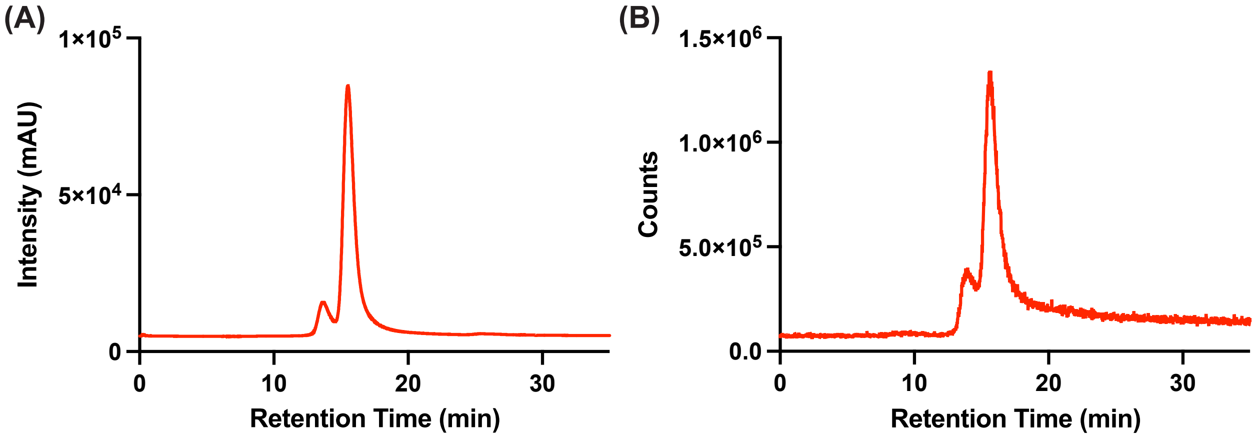

Figure S8. (A) UV-Vis (280 nm) and (B) radioactivity size exclusion radio-HPLC chromatograms of [^{89}Zr]Zr-DFO-[^{177}Lu]Lu-DOTA-^{TzAz}A33.

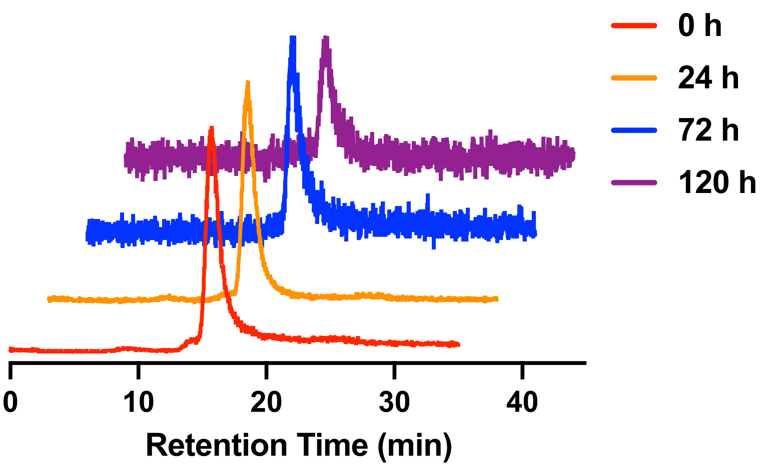

Figure S9. Radioactivity size-exclusion radio-HPLC chromatograms of [^{89}Zr]Zr-DFO-^{TzAz}A33 over five days.

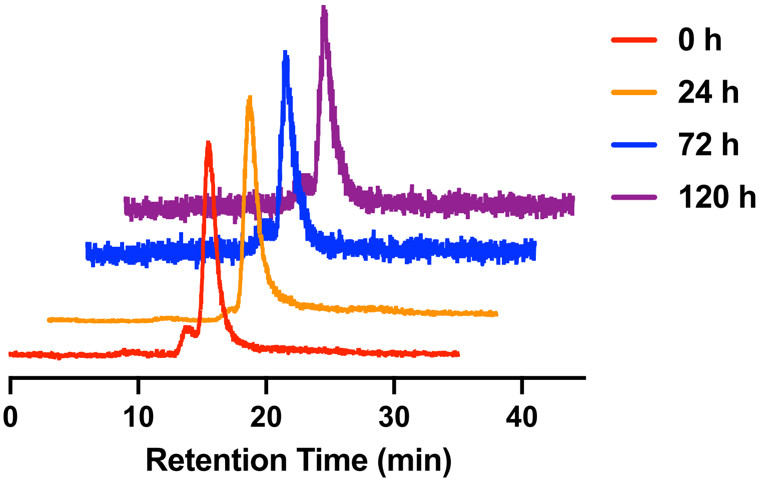

Figure S10. Radioactivity size-exclusion radio-HPLC chromatograms of [^{89}Zr]Zr-DFO-DOTA-^{TzAz}A33 over five days.

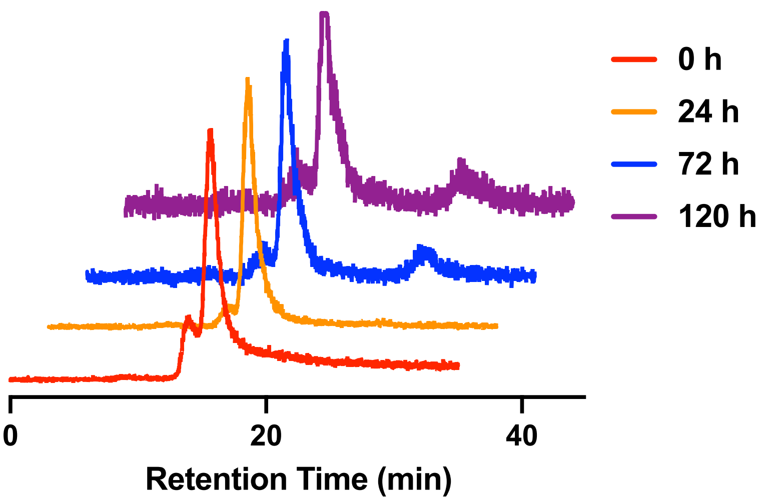

Figure S11. Radioactivity size-exclusion radio-HPLC chromatograms of [^{89}Zr]Zr-DFO-[^{177}Lu]Lu-DOTA-^{TzAz}A33 over five days.

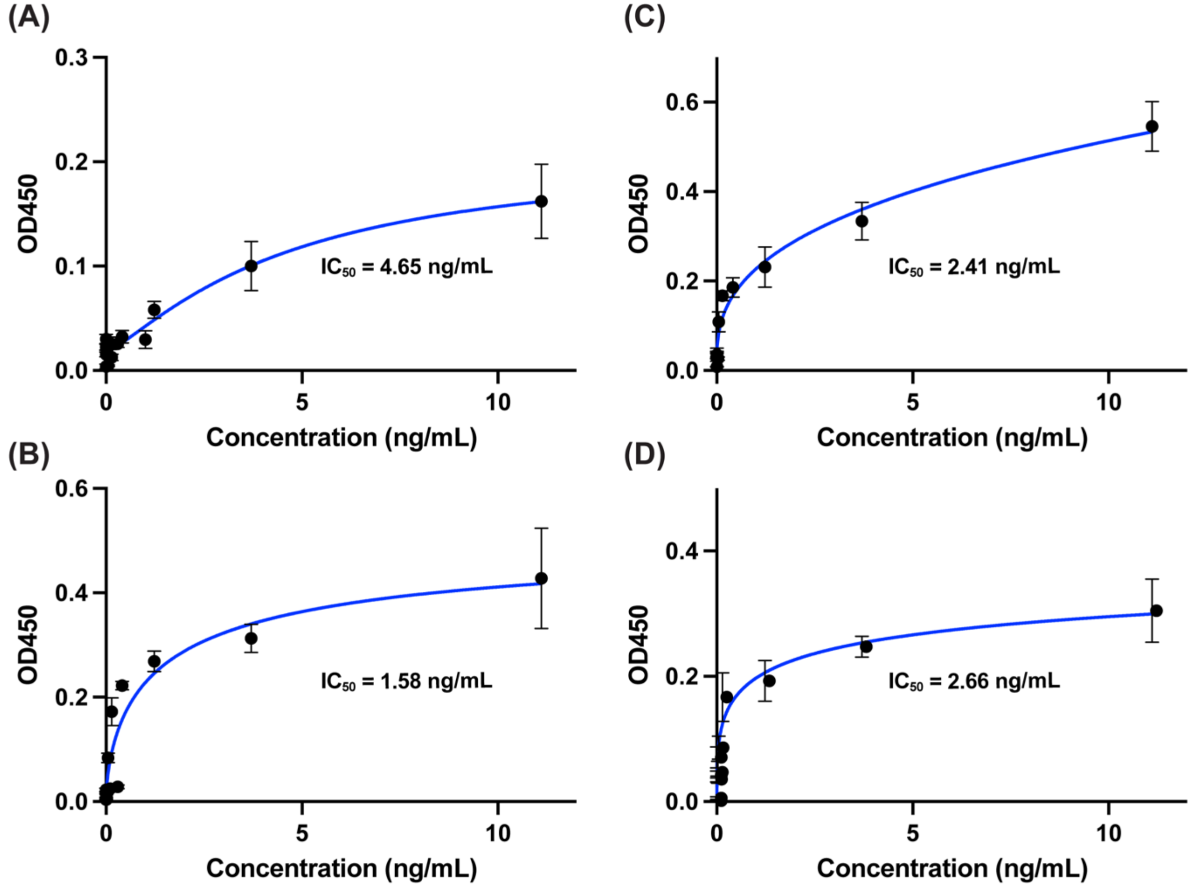

Figure S12. ELISA analysis of each immunoconjugates (A) A33 (B) ^{TzAz}A33 (C) DFO-^{TzAz}A33 (D) DFO-DOTA-^{TzAz}A33.

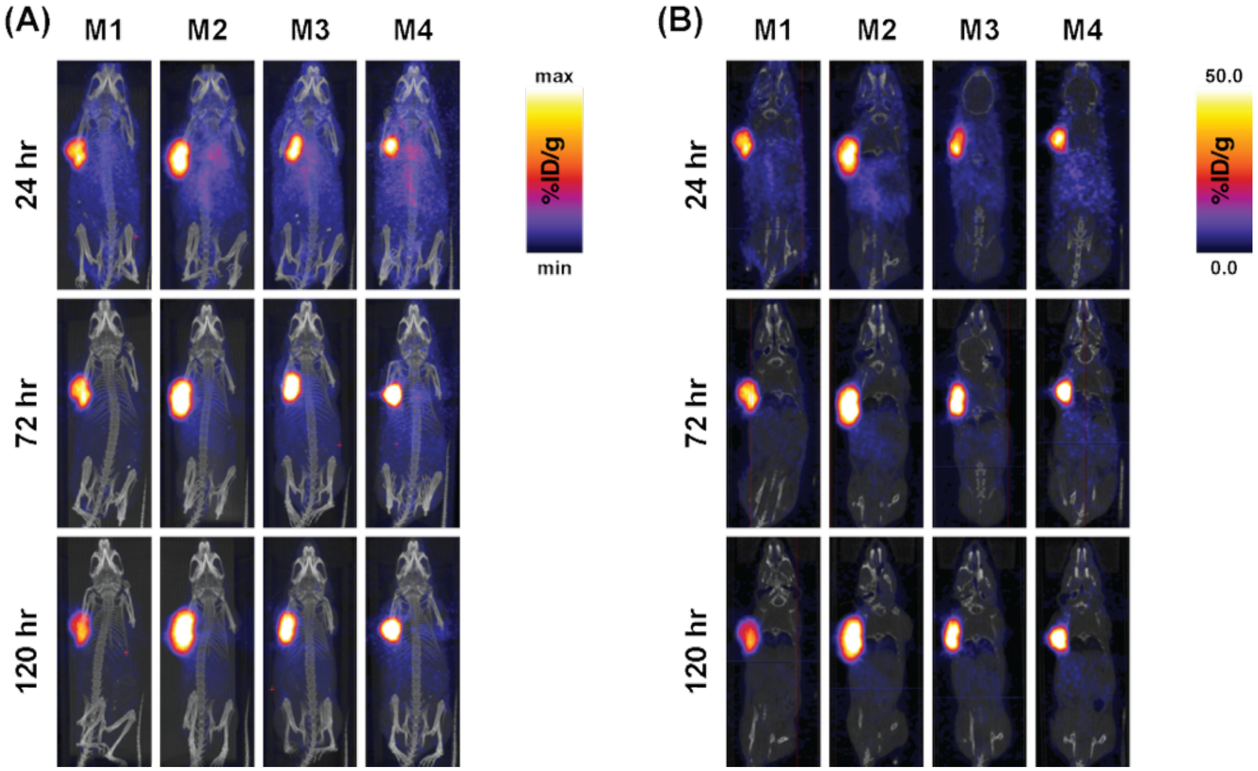

Figure S13. Maximum intensity projection (A) and coronal slice (B) PET images acquired 24, 72, and 120 h after the administration of [^{89}Zr]Zr-DFO-^{TzAz}A33 [100 μCi (3.7 MBq), 33.0 μg in 100 μL of PBS] to athymic nude mice bearing SW1222 xenografts (n = 4). (A) These data correspond with that described in *Table S4*.

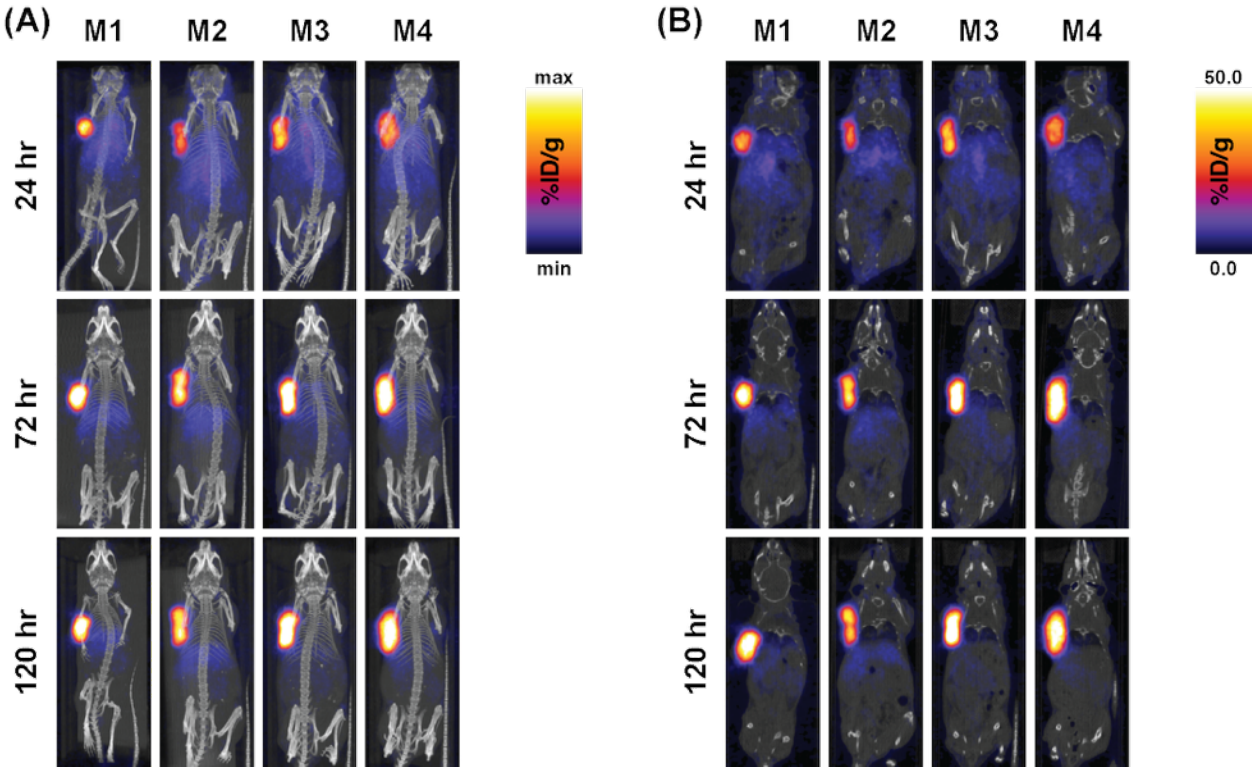

Figure S14. Maximum intensity projection (A) and coronal slice (B) PET images acquired 24, 72, and 120 h after the administration of [^{89}Zr]Zr-DFO-DOTA-^{TzAz}A33 [100 µCi (3.7 MBq), 33.0 µg in 100 µL of PBS] to athymic nude mice bearing SW1222 xenografts (n = 4). These data correspond with that described in *Table S4*.

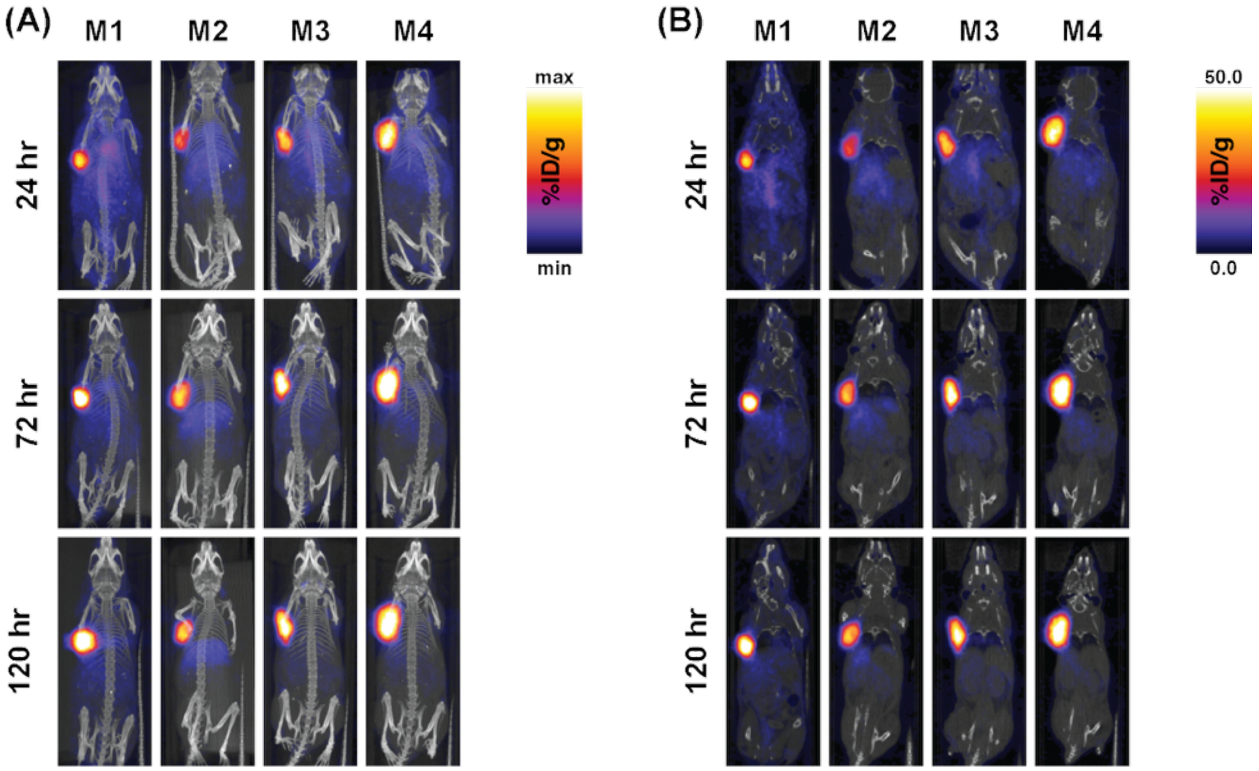

Figure S15. Maximum intensity projection (A) and coronal slice (B) PET images acquired 24, 72, and 120 h after the administration of [^{89}Zr]Zr-DFO-[^{177}Lu]Lu-DOTA-^{TzAz}A33 [100 µCi (3.7 MBq), 33.0 µg in 100 µL of PBS] to athymic nude mice bearing SW1222 xenografts (n = 4). These data correspond with that described in *Table S5*.

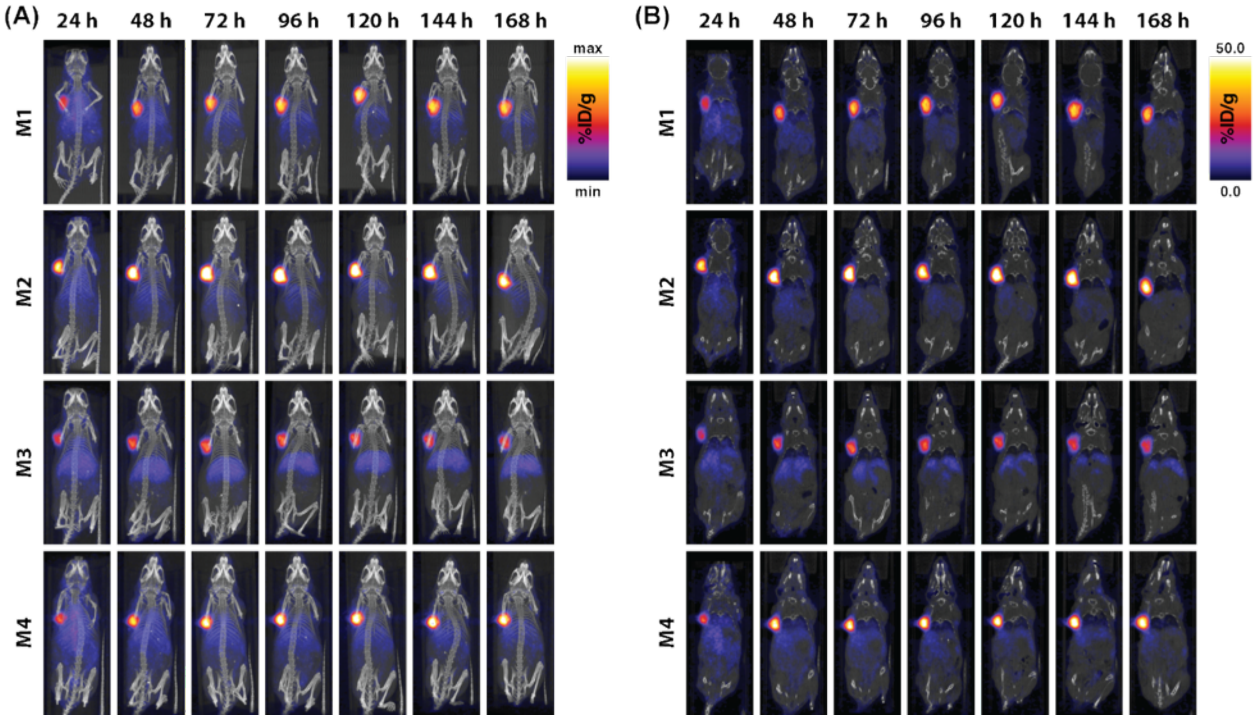

Figure S16. Maximum intensity projection (A) and coronal slice (B) PET images acquired 24, 48, 72, 96, 120, 144, or 168 h after the administration of [^{89}Zr]Zr-DFO-[^{177}Lu]Lu-DOTA-^{TzAz}A33 [100 μCi (3.7 MBq), 33.0 μg, in 100 μL of PBS] to athymic nude mice bearing SW1222 xenografts (n = 4 per time point). These data correspond with that described in *Figure 5* and *Tables S6–S9*.

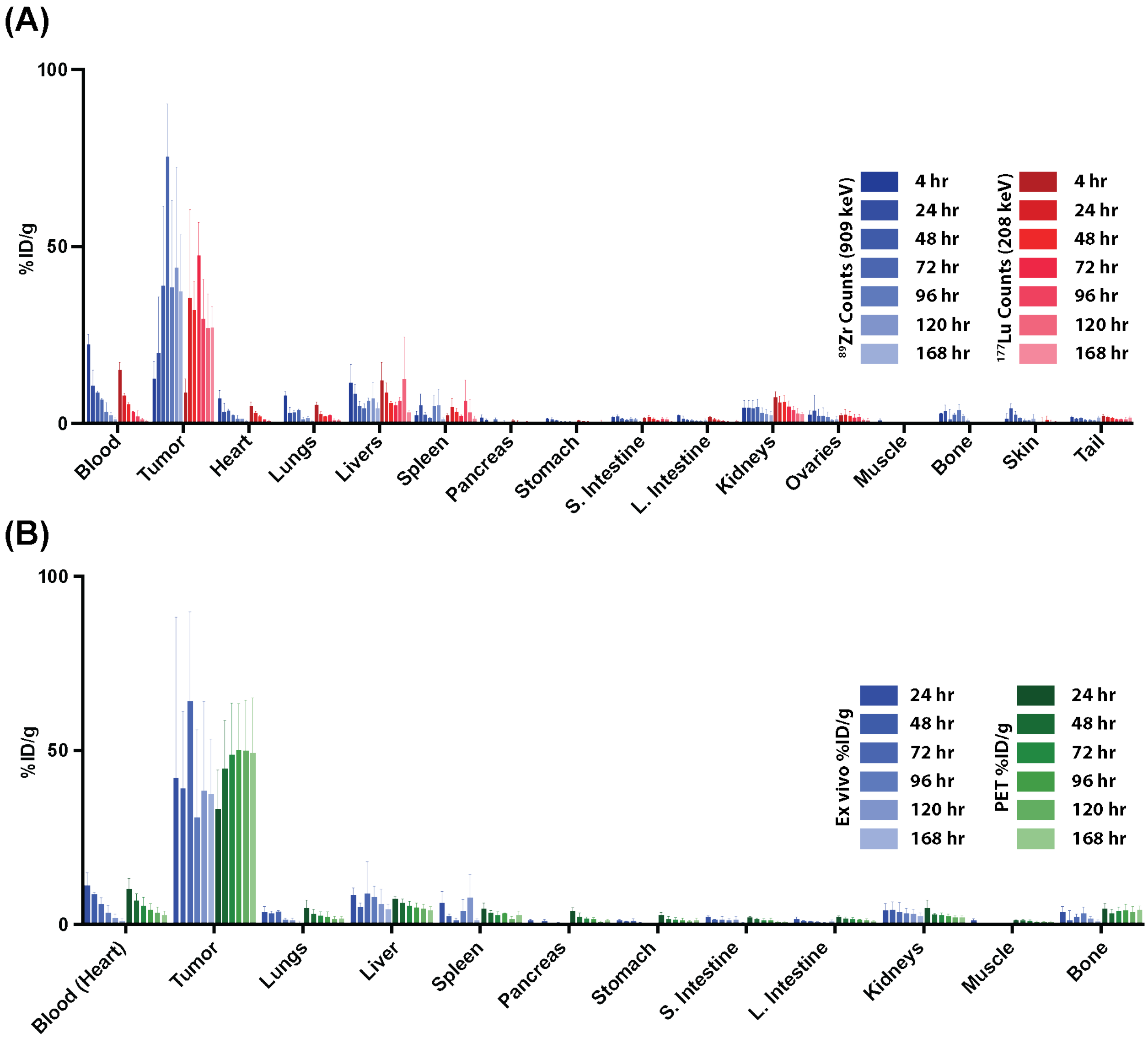

Figure S17. (A) Biodistribution data acquired 24, 48, 72, 96, 120, and 168 h after the administration of [^{89}Zr]Zr-DFO-[^{177}Lu]Lu-DOTA-^{TzAz}A33 [100 µCi (3.7 MBq), 33.0 µg, in 100 µL of PBS] to athymic nude mice bearing SW1222 xenografts (n = 4 per time point). ^{89}Zr counts are shown in blue, and ^{177}Lu counts are shown in red. These data correspond with that described in *Figure 5B* and *Tables S6–S7*. (B) Comparison of ^{89}Zr biodistribution data obtained via *ex vivo* biodistribution (blue) and ^{89}Zr biodistribution data acquired via PET image analysis (green). These data correspond with that described in *Figure 5C* and *Tables S8-S9*.

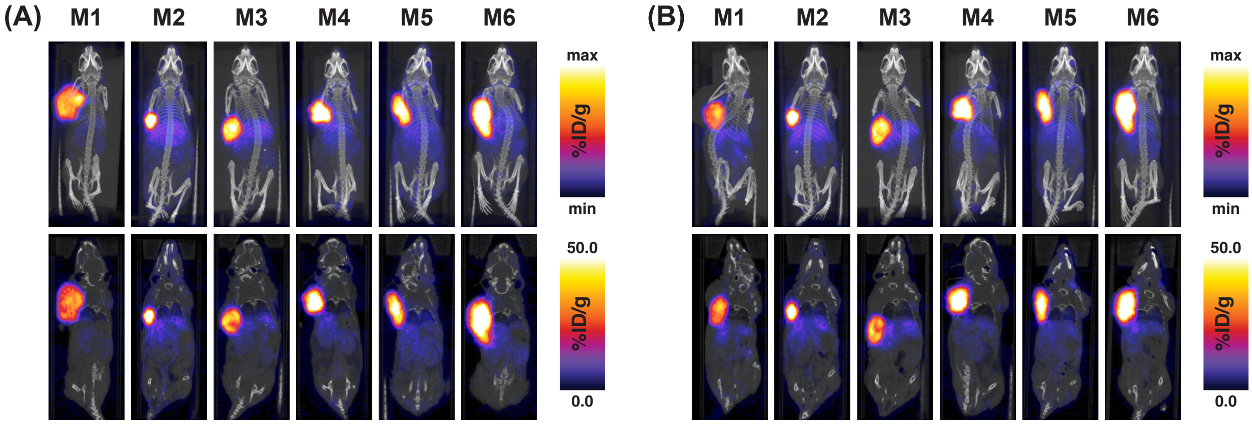

Figure S18. Maximum intensity projection (top) and coronal slice (bottom) PET images acquired 48 (A) and 72 h (B) after the administration of [^{89}Zr]Zr-DFO-[^{177}Lu]Lu-DOTA-^{TzAz}A33 [100 µCi (3.7 MBq) ^{89}Zr and 200 µCi (7.4 MBq) ^{177}Lu; 33.0 µg in 100 µL of PBS] to athymic nude mice bearing SW1222 xenografts (M1-M6 within the longitudinal therapy study).

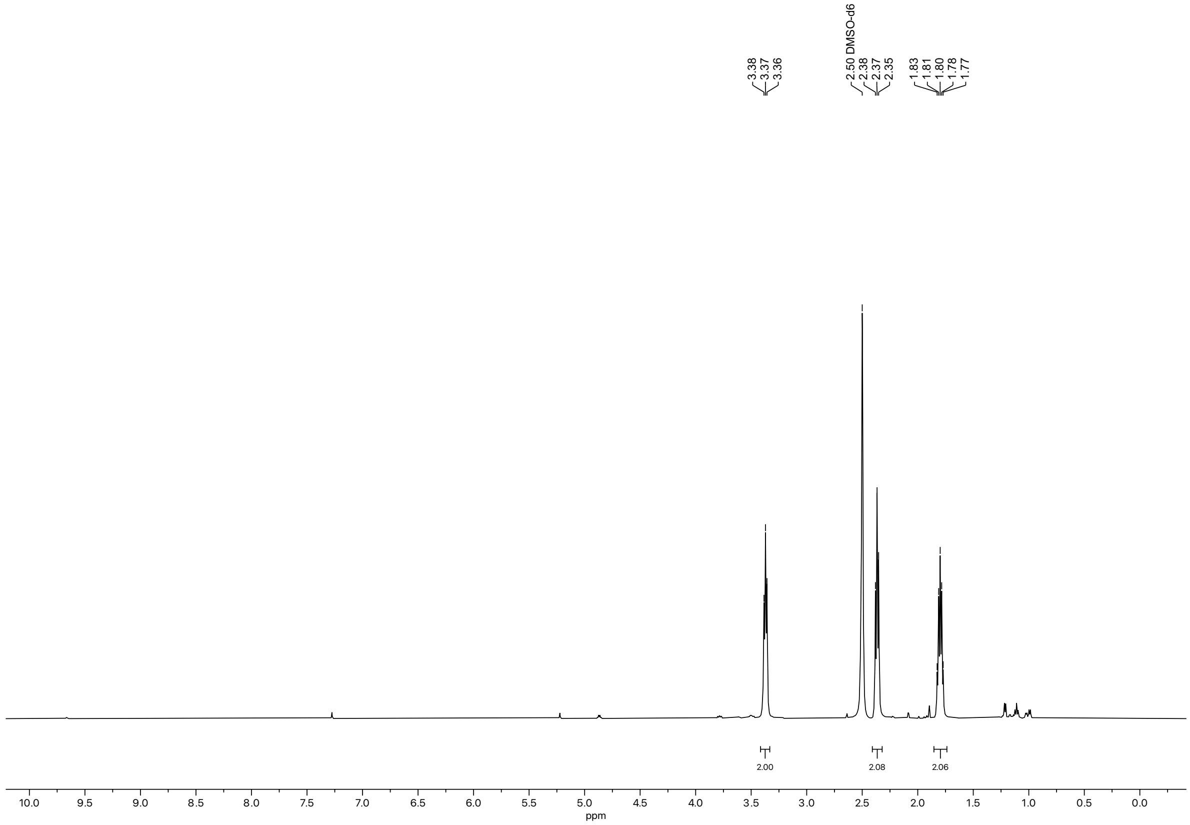

Figure S19. ^{1}H-NMR spectra of Az-CN

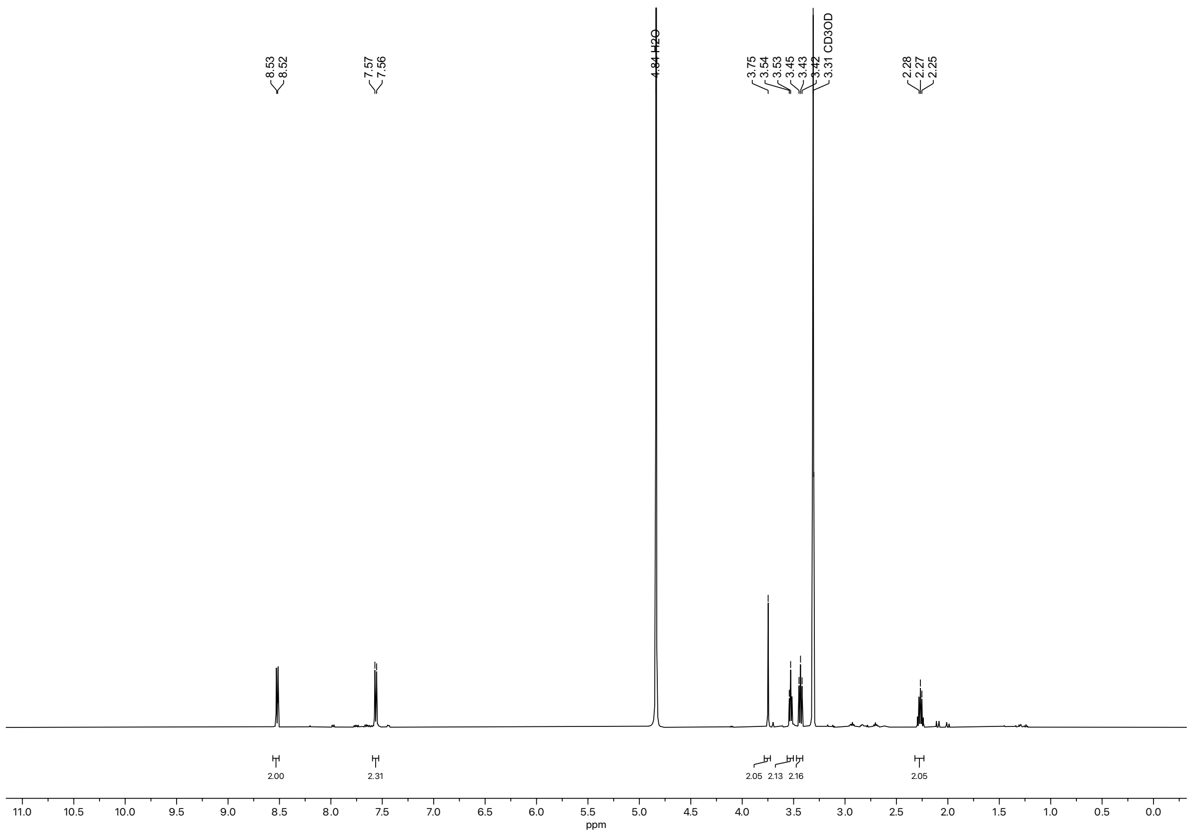

Figure S20. ^{1}H-NMR spectra of TzAz-COOH

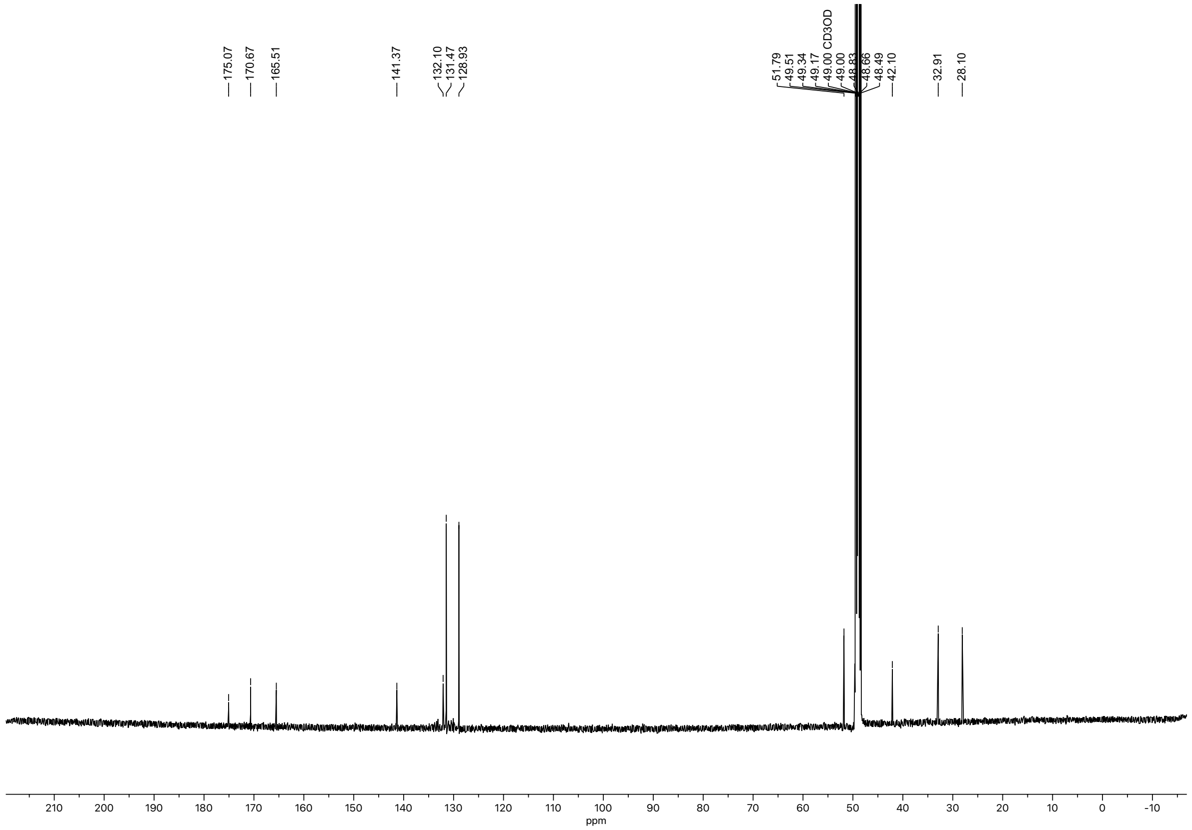

Figure S21. ^{13}C-NMR spectra of TzAz-COOH

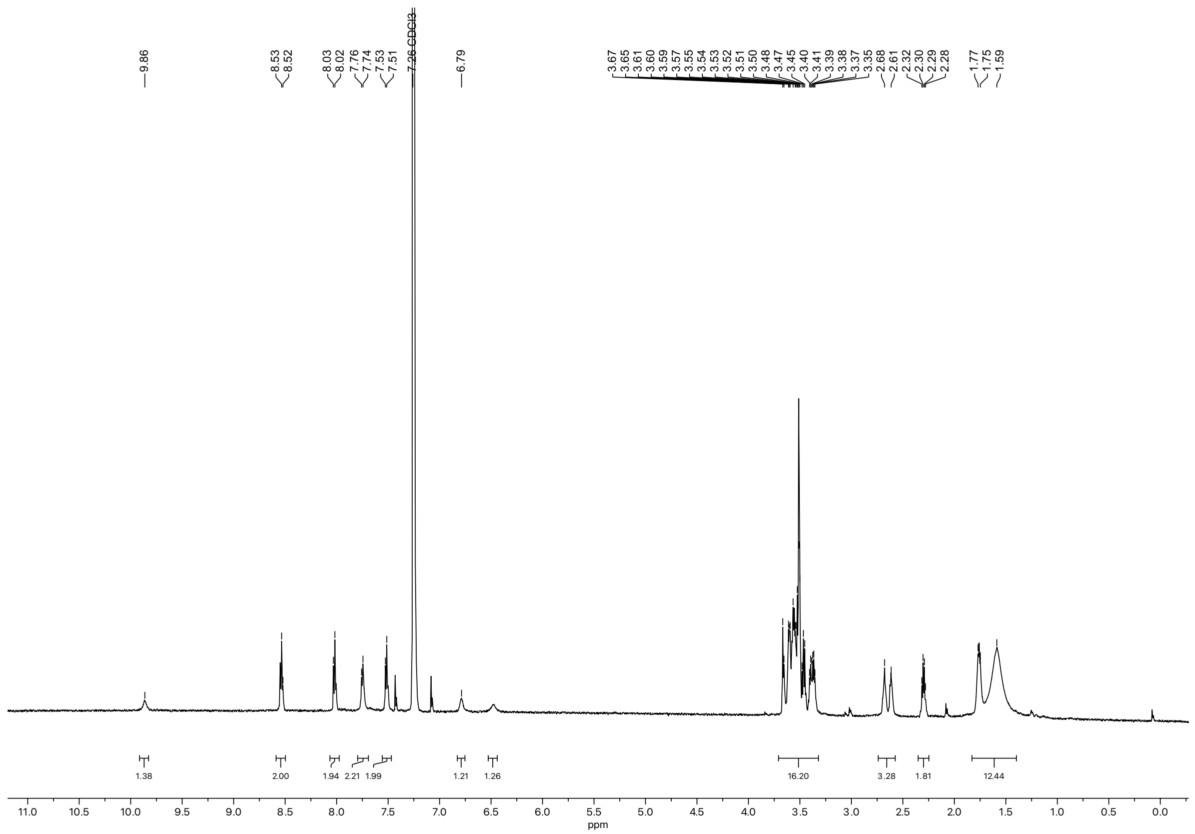

Figure S22. ^{1}H-NMR spectra of TzAz-PODS

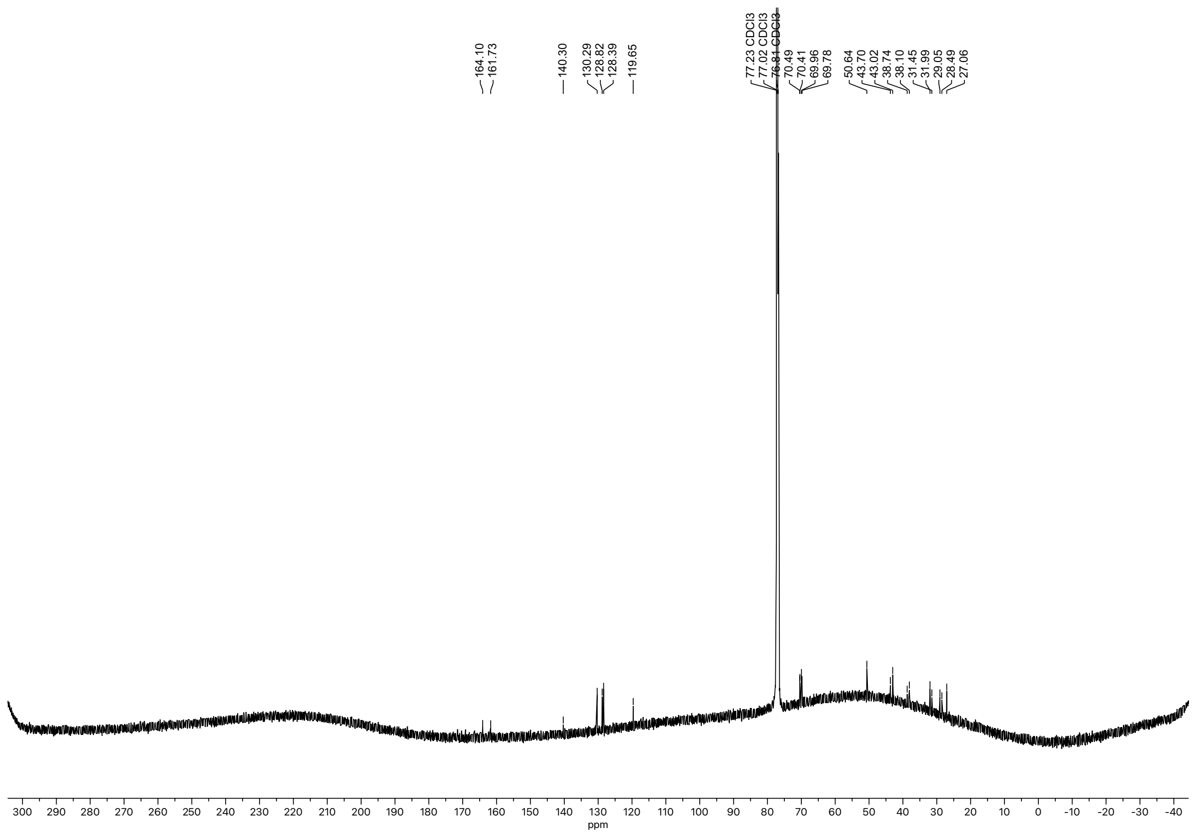

Figure S23. ^{13}C-NMR spectra of TzAz-PODS

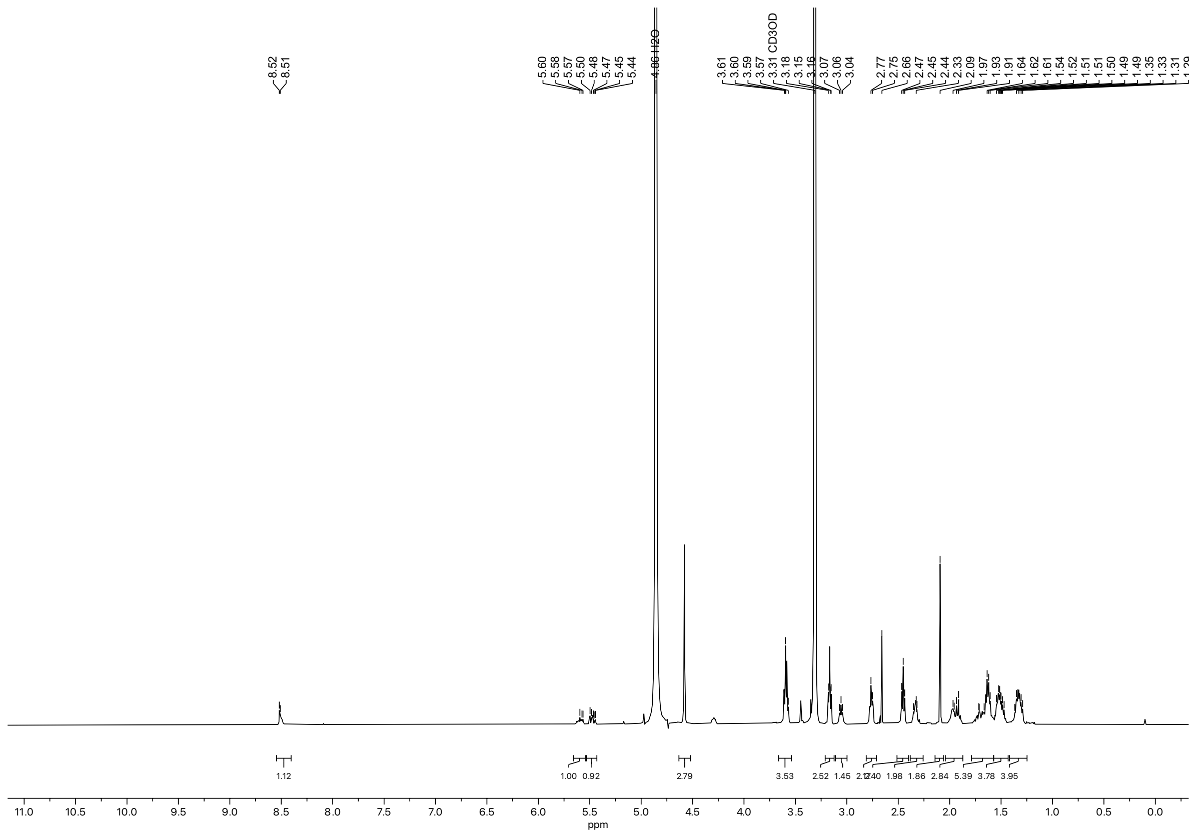

Figure S24. ^{1}H-NMR spectra of TCO-DFO

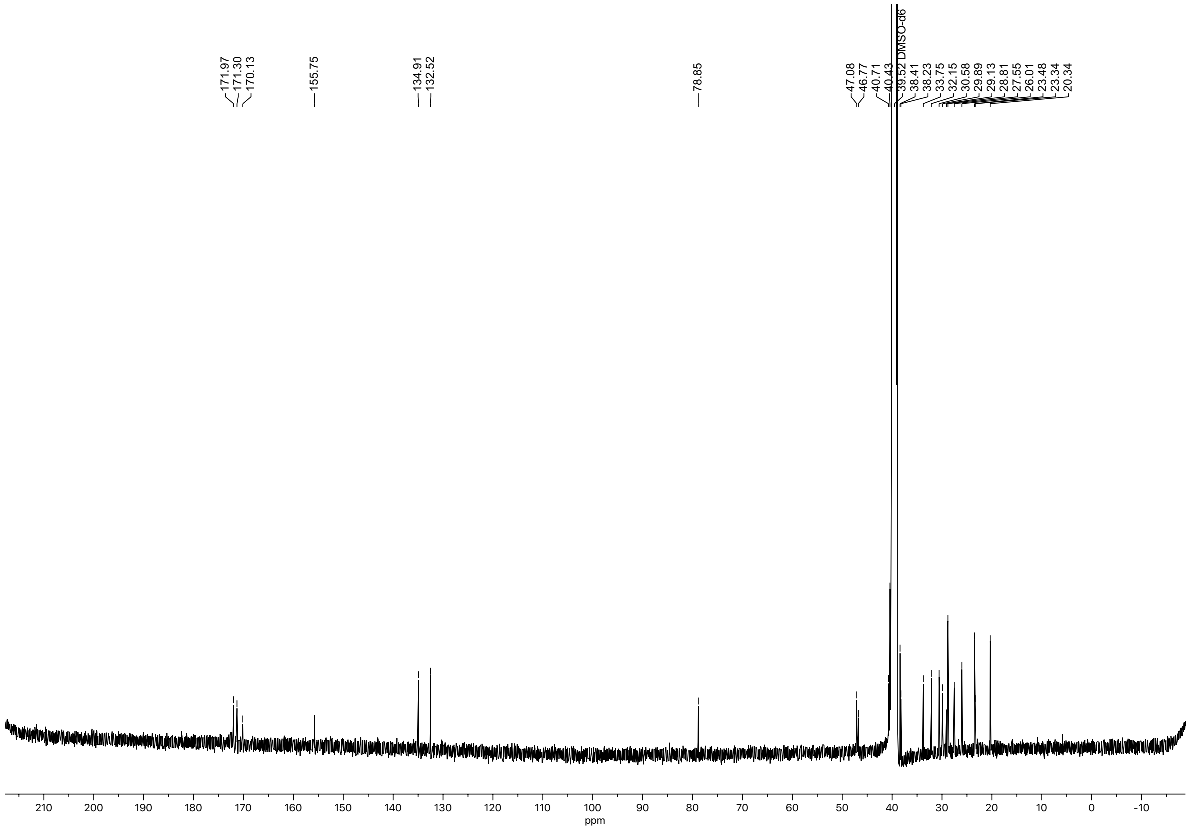

Figure S25. ^{13}C-NMR spectra of TCO-DFO

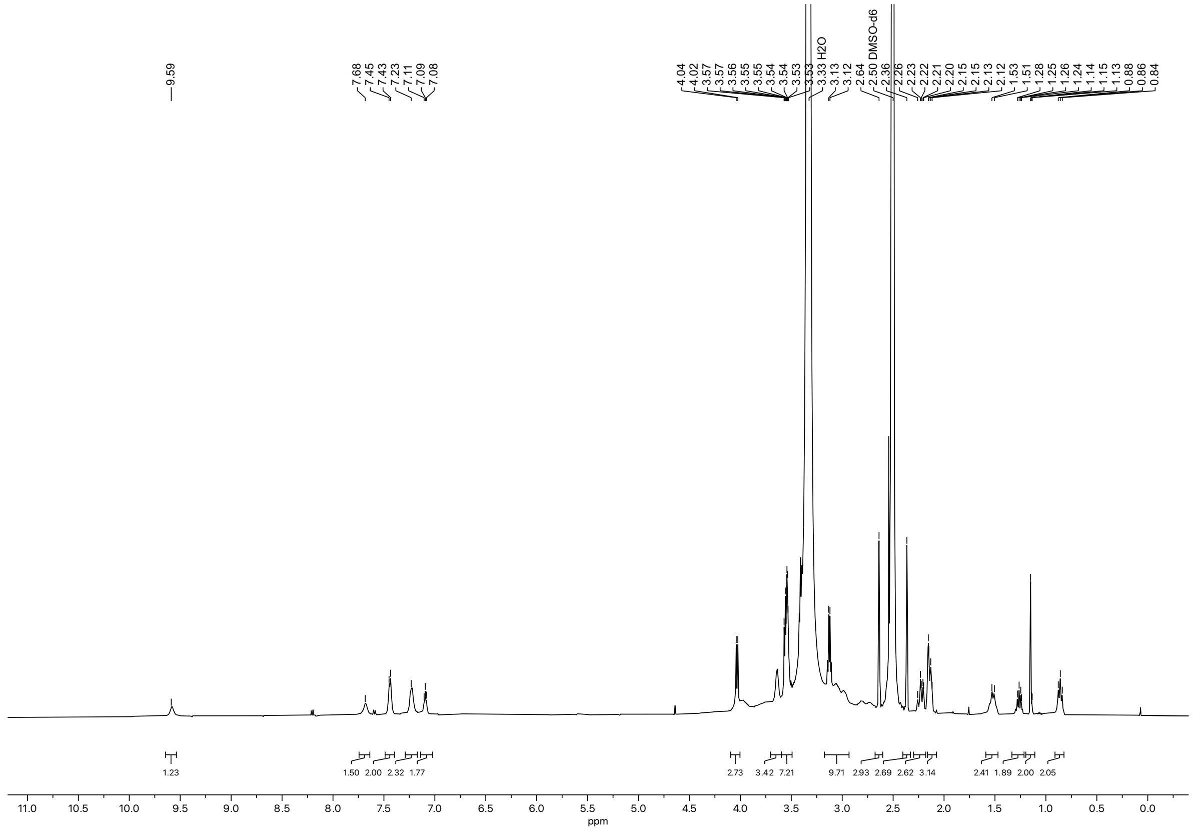

Figure S26. ^{1}H-NMR spectra of BCN-DOTA

Supplementary Tables and Table Legends

Sample	Average Mass (Da; n = 3)	DOL (cargo/mAb)
**5B1**	148370	–
**^{TzAz}5B1**	150260 ± 586	2.3 ± 0.1
**A33**	148964 ± 18	–
**^{TzAz}A33**	150867.3 ± 111	2.3 ± 0.2
**DFO-^{TzAz}A33**	152907.2 ± 26	2.6 ± 0.0
**DFO-DOTA-^{TzAz}A33**	155056.3 ± 674	2.6 ± 0.3

Table S1. MALDI-ToF mass spectrometry data used to determine the degree of labeling (DOL) of each immunoconjugate.

Sample	Cargo	
	**Cy5**	**AF488**
Cy5-^{TzAz}5B1	2.1 ± 0.1	–
AF488-^{TzAz}5B1	–	2.5 ± 0.3
Cy5/AF488-^{TzAz}5B1	2.1 ± 0.4	2.2 ± 0.5
Cy5- ^{TzAz}A33	2.4 ± 0.6	–
AF488- ^{TzAz}A33	–	4.0 ± 0.9
Cy5/AF488- ^{TzAz}A33	2.4 ± 0.2	2.3 ± 0.5

Table S2. UV-Vis spectrophotometry-derived DOLs for the fluorophore-bearing immunoconjugates

Radioimmunoconjugates	Specific Activity (mCi/mg)	Yield (%)	Purity (%)
[^{89}Zr]Zr-DFO- ^{TzAz}A33	3.0	98.5	99.3
[^{89}Zr]Zr-DFO-DOTA- ^{TzAz}A33	3.0	97.4	99.4
[^{89}Zr]Zr-DFO-[^{177}Lu]Lu-DOTA-^{TzAz}A33	3.0 (^{89}Zr); 3.0 (^{177}Lu)	98.9	99.3

Table S3. Radiolabeling data for each radioimmunoconjugate, including specific activity, radiochemical yield, and purity.

Tissue	[^{89}Zr]Zr-DFO-^{TzAz}A33	[^{89}Zr]Zr-DFO-DOTA-^{TzAz}A33
**Blood**	1.8 ± 0.8	2.3 ± 0.7
**Tumor**	27.6 ± 11.5	26.4 ± 8.9
**Heart**	1.2 ± 0.3	1.1 ± 0.2
**Lungs**	1.1 ± 0.6	1.1 ± 0.4
**Liver**	2.2 ± 0.7	4.2 ± 1.6
**Spleen**	1.2 ± 0.7	1.3 ± 1.1
**Pancreas**	0.4 ± 0.2	0.2 ± 0.1
**Stomach**	0.4 ± 0.2	0.3 ± 0.1
**S. Intestine**	0.7 ± 0.1	0.7 ± 0.2
**L. Intestine**	0.7 ± 0.1	0.5 ± 0.1
**Kidneys**	1.6 ± 0.7	1.2 ± 0.2
**Ovaries**	0.9 ± 0.6	1.6 ± 1.2
**Muscle**	0.2 ± 0.03	0.2 ± 0.05
**Bone**	2.0 ± 0.5	1.4 ± 1.0
**Skin**	1.0 ± 0.4	1.3 ± 0.3
**Tail**	0.8 ± 0.3	1.2 ± 0.3

Table S4. Biodistribution data collected 120 h after the intravenous administration of [^{89}Zr]Zr-DFO-^{TzAz}A33 or [^{89}Zr]Zr-DFO-DOTA-^{TzAz}A33 [100 µCi (3.7 MBq) in 100 µL of PBS] to SW1222 xenografted athymic nude mice. Values are in units of %ID/g and are expressed as mean ± standard deviation. The data corresponds with data shown in *Figures 4A, 4B, S5, and S6.*

Tissue	[^{89}Zr]Zr-DFO-[^{177}Lu]Lu-DOTA-^{TzAz}A33	
	^{89}Zr Counts (909 keV)	^{177}Lu Counts* (208 keV)
**Blood**	2.3 ± 1.5	0.9 ± 0.4
**Tumor**	38.7 ± 14.1	28.4 ± 6.1
**Heart**	0.4 ± 0.1	0.0 ± 0.3
**Lungs**	0.4 ± 0.5	0.4 ± 0.2
**Liver**	5.3 ± 2.0	3.0 ± 0.3
**Spleen**	1.2 ± 0.3	1.4 ± 0.6
**Pancreas**	0.3 ± 0.1	0.3 ± 0.2
**Stomach**	0.4 ± 0.1	0.7 ± 0.7
**S. Intestine**	0.5 ± 0.2	0.7 ± 0.9
**L. Intestine**	0.7 ± 0.2	0.6 ± 0.2
**Kidneys**	2.5 ± 1.8	2.8 ± 0.7
**Ovaries**	1.1 ± 0.2	1.4 ± 0.5
**Muscle**	0.4 ± 0.1	-0.2 ± 0.2‡
**Bone**	0.7 ± 0.3	-1.2 ± 0.9‡
**Skin**	1.3 ± 0.3	-0.4 ± 0.2‡
**Tail**	1.1 ± 0.1	1.5 ± 0.7

Table S5. Biodistribution data collected 120 h after the intravenous administration of [^{89}Zr]Zr-DFO-[^{177}Lu]Lu-DOTA-^{TzAz}A33 [100 µCi (3.7 MBq) of ^{89}Zr and 100 µCi (3.7 MBq) of ^{177}Lu in 100 µL of PBS] to SW1222 xenografted athymic nude mice. ^{89}Zr and ^{177}Lu radioactive counts were acquired at 909 keV and 208 keV, respectively. Values are in units of %ID/g and are expressed as mean ± standard deviation. The data corresponds with the data shown in *Figures 4C, and S7*. (*The actual ^{177}Lu counts were calibrated via the calibration line provided in the calibration method to remove the background counts caused by ^{89}Zr. ‡Below the limit of detection for ^{177}Lu counts)

	^{89}Zr Counts (909 keV)						
	**4 h**	**24 h**	**48 h**	**72 h**	**96 h**	**120 h**	**168 h**
**Blood**	22.3 ± 2.8	10.7 ± 4.5	8.6 ± 0.6	6.8 ± 0.3	3.2 ± 2.6	2.3 ± 0.7	1.1 ± 0.6
**Tumor**	12.6 ± 5.0	19.8 ± 16.0	39.0 ± 22.4	75.3 ± 14.9	38.4 ± 24.6	43.9 ± 28.5	37.3 ± 16.1
**Heart**	7.0 ± 2.4	3.4 ± 2.4	3.7 ± 0.5	2.3 ± 0.2	1.3 ± 0.9	1.2 ± 0.1	0.6 ± 0.3
**Lungs**	7.9 ± 1.1	3.0 ± 1.5	3.1 ± 0.7	3.7 ± 0.4	1.2 ± 0.6	1.5 ± 0.4	0.6 ± 0.4
**Liver**	11.6 ± 5.1	8.4 ± 2.7	5.0 ± 1.2	4.2 ± 1.4	6.3 ± 0.8	6.9 ± 4.7	4.3 ± 1.7
**Spleen**	2.3 ± 1.2	5.1 ± 3.2	2.4 ± 0.7	1.5 ± 0.2	4.9 ± 3.1	5.0 ± 4.6	1.1 ± 0.6
**Pancreas**	1.7 ± 0.8	1.0 ± 0.4	0.5 ± 0.1	1.1 ± 0.5	0.3 ± 0.1	0.4 ± 0.3	0.2 ± 0.1
**Stomach**	1.3 ± 0.2	1.2 ± 0.5	0.8 ± 0.2	0.6 ± 0.1	0.5 ± 0.2	0.4 ± 0.1	0.3 ± 0.2
**S. Intestine**	1.9 ± 0.3	2.0 ± 0.5	1.3 ± 0.2	0.9 ± 0.2	1.3 ± 0.5	1.0 ± 0.3	0.5 ± 0.2
**L. Intestine**	2.3 ± 0.2	1.4 ± 0.8	1.0 ± 0.2	0.8 ± 0.3	0.7 ± 0.1	0.5 ± 0.1	0.8 ± 0.5
**Kidneys**	4.4 ± 2.2	4.5 ± 2.0	4.2 ± 2.3	4.5 ± 2.4	2.9 ± 1.6	2.5 ± 1.4	2.3 ± 1.1
**Ovaries**	2.4 ± 1.2	3.7 ± 4.3	2.1 ± 2.0	2.2 ± 2.3	1.7 ± 1.5	1.0 ± 0.1	1.2 ± 0.8
**Muscle**	0.4 ± 0.1	0.9 ± 0.4	0.3 ± 0.1	0.2 ± 0.02	0.2 ± 0.1	0.1 ± 0.1	0.2 ± 0.1
**Bone**	2.7 ± 0.1	3.4 ± 1.9	1.2 ± 2.8	2.5 ± 0.4	3.8 ± 1.7	2.0 ± 0.5	0.9 ± 0.4
**Skin**	1.3 ± 1.4	4.2 ± 1.3	2.4 ± 1.0	1.4 ± 0.3	0.9 ± 0.4	0.8 ± 0.05	1.2 ± 1.3
**Tail**	1.8 ± 0.4	1.4 ± 0.1	1.4 ± 0.2	1.1 ± 0.2	0.9 ± 0.2	0.8 ± 0.1	1.6 ± 0.4

Table S6. ^{89}Zr biodistribution data collected 4, 24, 48, 72, 96, 120, 168 h (n = 4 for each time point) after the intravenous administration of [^{89}Zr]Zr-DFO-[^{177}Lu]Lu-DOTA-^{TzAz}A33 [100 μCi (3.7 MBq) of ^{89}Zr and 100 μCi (3.7 MBq) of ^{177}Lu in 100 μL of PBS] to SW1222 xenografted athymic nude mice. ^{89}Zr radioactive counts were acquired at 909 keV. Values are in units of %ID/g and are expressed as mean ± standard deviation. The data corresponds with the data shown in *Figures 5 and S8*. (*The actual ^{177}Lu counts were calibrated via the calibration line in the calibration method to remove the background counts caused by ^{89}Zr.)

	^{177}Lu Counts* (208 keV)						
	**4 h**	**24 h**	**48 h**	**72 h**	**96 h**	**120 h**	**168 h**
**Blood**	15.1 ± 2.1	8.0 ± 0.6	5.5 ± 0.4	3.4 ± 0.1	1.9 ± 1.7	1.2 ± 0.3	0.7 ± 0.3
**Tumor**	8.8 ± 3.9	35.4 ± 25.0	32.0 ± 8.0	47.4 ± 9.3	29.5 ± 11.2	26.9 ± 9.8	27.1 ± 5.8
**Heart**	5.0 ± 1.2	2.9 ± 0.5	2.0 ± 0.3	1.1 ± 0.1	0.6 ± 0.4	0.4 ± 0.1	0.04 ± 0.2
**Lungs**	5.3 ± 0.8	2.7 ± 0.8	2.0 ± 0.2	2.2 ± 0.2	0.9 ± 0.5	0.9 ± 0.2	0.3 ± 0.2
**Liver**	12.1 ± 5.2	8.7 ± 2.7	5.8 ± 0.5	5.1 ± 1.0	6.5 ± 1.0	12.5 ± 12.0	3.1 ± 0.6
**Spleen**	2.3 ± 0.5	4.6 ± 2.5	3.3 ± 1.0	2.1 ± 0.4	6.3 ± 5.9	3.1 ± 3.6	1.3 ± 0.8
**Pancreas**	0.8 ± 0.3	0.5 ± 0.3	0.2 ± 0.1	0.4 ± 0.2	-0.03 ± 0.1‡	-0.02 ± 0.1‡	-0.1 ± 0.1‡
**Stomach**	0.8 ± 0.2	0.6 ± 0.1	0.5 ± 0.1	0.4 ± 0.1	0.2 ± 0.1	0.5 ± 0.5	0.2 ± 0.1
**S. Intestine**	1.5 ± 0.3	1.8 ± 0.6	1.3 ± 0.3	0.8 ± 0.2	1.3 ± 0.6	1.1 ± 0.5	0.5 ± 0.2
**L. Intestine**	1.8 ± 0.2	1.2 ± 0.2	0.8 ± 0.2	0.7 ± 0.2	0.6 ± 0.1	0.4 ± 0.2	0.7 ± 0.3
**Kidneys**	7.4 ± 1.7	5.9 ± 1.9	6.1 ± 1.8	4.8 ± 1.6	3.8 ± 1.1	2.8 ± 1.5	2.6 ± 0.5
**Ovaries**	2.2 ± 0.5	2.4 ± 1.5	2.2 ± 1.2	1.6 ± 1.0	1.8 ± 0.8	0.9 ± 0.5	0.9 ± 0.5
**Muscle**	-1.0 ± 0.6‡	-0.8 ± 1.0‡	-0.7 ± 0.3‡	-0.8 ± 0.2‡	-0.9 ± 0.7‡	-0.8 ± 0.4‡	-0.4 ± 0.1‡
**Bone**	-2.8 ± 1.7‡	-5.7 ± 5.6‡	-3.3 ± 3.4‡	-4.1 ± 0.4‡	-2.8 ± 2.9‡	-3.6 ± 1.4‡	-2.7 ± 1.1‡
**Skin**	-0.7 ± 1.0‡	0.4 ± 1.2	0.9 ± 1.3	-0.03 ± 1.0‡	-1.1 ± 1.7‡	-0.2 ± 0.4‡	-0.3 ± 0.2‡
**Tail**	2.1 ± 0.5	1.7 ± 0.5	1.5 ± 0.3	1.1 ± 0.2	1.1 ± 0.2	1.1 ± 0.7	1.7 ± 0.4

Table S7. ^{177}Lu biodistribution data collected 4, 24, 48, 72, 96, 120, 168 h (n = 4 for each time point) after the intravenous administration of [^{89}Zr]Zr-DFO-[^{177}Lu]Lu-DOTA-^{TzAz}A33 [100 µCi (3.7 MBq) of ^{89}Zr and 100 µCi (3.7 MBq) of ^{177}Lu in 100 µL of PBS] to SW1222 xenografted athymic nude mice. ^{177}Lu radioactive counts were acquired at 208 keV. Values are in units of %ID/g and are expressed as mean ± standard deviation. The data corresponds with the data shown in Figures *5 and S8*. (*The actual ^{177}Lu counts were calibrated via the calibration line in the method section to remove the background counts caused by ^{89}Zr. ‡Below the limit of detection for ^{177}Lu counts)

Tissue	$[^{89}Zr]Zr$-DFO-$[^{177}Lu]Lu$-DOTA-^{TzAz}A33					
	24 h	48 h	72 h	96 h	120 h	168 h
**Blood**	11.1 ± 3.8	8.6 ± 0.6	5.9 ± 1.8	3.3 ± 2.1	1.8 ± 1.2	1.1 ± 0.6
**Tumor**	42.0 ± 46.3	39.0 ± 22.4	64.0 ± 25.7	20.7 ± 25.2	38.3 ± 25.8	37.3 ± 16.1
**Lungs**	3.5 ± 1.6	3.1 ± 0.7	3.6 ± 0.3	1.3 ± 0.5	1.2 ± 0.6	0.6 ± 0.4
**Liver**	8.3 ± 2.2	5.0 ± 1.2	8.8 ± 9.2	7.9 ± 3.2	5.8 ± 4.4	4.3 ± 1.7
**Spleen**	6.2 ± 3.4	2.4 ± 0.7	1.2 ± 0.6	3.9 ± 3.2	7.7 ± 6.6	1.1 ± 0.6
**Pancreas**	1.1 ± 0.4	0.5 ± 0.1	1.0 ± 0.5	0.3 ± 0.1	0.4 ± 0.3	0.2 ± 0.1
**Stomach**	1.1 ± 0.4	0.8 ± 0.2	0.9 ± 0.7	0.5 ± 0.2	0.4 ± 0.2	0.3 ± 0.1
**S. Intestine**	2.1 ± 0.5	1.3 ± 0.2	1.4 ± 0.9	1.2 ± 0.5	1.4 ± 0.9	0.5 ± 0.3
**L. Intestine**	1.5 ± 0.7	1.0 ± 0.2	0.8 ± 0.3	0.6 ± 0.1	0.6 ± 0.1	0.8 ± 0.5
**Kidneys**	4.0 ± 1.9	4.2 ± 2.3	3.6 ± 2.7	3.2 ± 1.4	3.0 ± 1.4	2.3 ± 1.1
**Muscle**	1.1 ± 0.5	0.3 ± 0.1	0.2 ± 0.1	0.1 ± 0.1	0.1 ± 0.1	0.2 ± 0.1
**Bone**	3.5 ± 1.6	1.2 ± 2.8	2.1 ± 0.9	3.1 ± 1.9	1.7 ± 0.7	0.9 ± 0.4

Table S8. PET-derived ^{89}Zr biodistribution data acquired via imaging 4, 24, 48, 72, 96, 120, 168 h (n = 4 for each time point) after the intravenous administration of $[^{89}Zr]Zr$-DFO-$[^{177}Lu]Lu$-DOTA-^{TzAz}A33 [100 μCi (3.7 MBq) of ^{89}Zr and 100 μCi (3.7 MBq) of ^{177}Lu in 100 μL of PBS] to SW1222 xenografted athymic nude mice. Values are in units of %ID/g and are expressed as mean ± standard deviation.

Tissue	[^{89}Zr]Zr-DFO-[^{177}Lu]Lu-DOTA-^{TzAz}A33					
	**24 h**	**48 h**	**72 h**	**96 h**	**120 h**	**168 h**
**Blood**	10.2 ± 3.0	6.9 ± 2.0	5.4 ± 2.4	4.1 ± 1.9	3.4 ± 1.6	2.7 ± 1.2
**Tumor**	33.0 ± 11.4	44.7 ± 13.8	48.7 ± 14.9	50.0 ± 13.4	49.8 ± 14.6	49.2 ± 15.9
**Lungs**	4.7 ± 2.4	3.0 ± 1.3	2.5 ± 1.2	2.1 ± 1.4	1.6 ± 0.7	1.6 ± 0.8
**Liver**	7.3 ± 0.8	6.2 ± 1.1	5.3 ± 1.2	4.9 ± 1.2	4.6 ± 1.2	4.0 ± 1.1
**Spleen**	4.5 ± 1.7	3.4 ± 0.7	2.7 ± 1.0	3.1 ± 0.2	1.6 ± 0.8	2.6 ± 1.2
**Pancreas**	3.8 ± 1.1	2.2 ± 1.0	1.7 ± 0.4	1.5 ± 0.5	0.7 ± 0.3	1.2 ± 0.2
**Stomach**	2.6 ± 0.8	1.6 ± 0.8	1.4 ± 0.7	1.2 ± 0.7	0.9 ± 0.3	1.2 ± 0.6
**S. Intestine**	2.0 ± 0.3	1.4 ± 0.3	1.1 ± 0.6	1.1 ± 0.7	0.7 ± 0.2	0.7 ± 0.3
**L. Intestine**	2.2 ± 0.3	1.6 ± 0.5	1.6 ± 0.4	1.3 ± 0.2	1.2 ± 0.5	0.9 ± 0.3
**Kidneys**	4.7 ± 2.3	2.8 ± 0.5	2.6 ± 0.6	2.4 ± 0.6	1.9 ± 0.6	1.9 ± 0.6
**Muscle**	1.2 ± 0.04	1.1 ± 0.4	1.0 ± 0.4	0.7 ± 0.3	0.7 ± 0.2	0.6 ± 0.3
**Bone**	4.6 ± 1.5	3.2 ± 1.1	3.8 ± 1.3	4.0 ± 1.9	3.6 ± 1.6	4.1 ± 1.3

Table S9. *Ex vivo* ^{89}Zr biodistribution data collected 4, 24, 48, 72, 96, 120, 168 h (n = 4 for each time point) after the intravenous administration of [^{89}Zr]Zr-DFO-[^{177}Lu]Lu-DOTA-^{TzAz}A33 [100 µCi (3.7 MBq] of ^{89}Zr) in 100 µL of PBS] to SW1222 xenografted athymic nude mice. ^{89}Zr radioactive counts were acquired at 208 keV. Values are in units of %ID/g and are expressed as mean ± standard deviation. The data corresponds with data shown in *Figures 5C and S9*.

Tissue	^{177}Lu dose coefficients projected from ^{89}Zr Biodistribution [Gy/µCi]	^{177}Lu dose coefficients projected from ^{89}Zr PET [Gy/µCi]	^{177}Lu dose coefficients calculated from ^{177}Lu Biodistribution [Gy/µCi]
**Blood**	0.031	0.026	0.019
**Tumor**	0.271	0.284	0.177
**Heart**	0.012	N/A‡	0.006
**Lungs**	0.013	0.011	0.008
**Liver**	0.044	0.040	0.046
**Spleen**	0.017	0.020	0.018
**Pancreas**	0.003	0.003	0.001
**Stomach**	0.004	0.004	0.002
**S. Intestine**	0.007	0.007	0.006
**Colon**	0.007	0.006	0.005
**Kidneys**	0.021	0.021	0.025
**Carcass**	0.010	0.002	0.011
**Bone Marrow**	0.011	0.010	0.007
**Compact Bone**	0.011	0.012	N/A*
**Ovaries**	0.011	N/A‡	0.009

Table S10. Dosimetry values for [^{89}Zr]Zr-DFO-[^{177}Lu]Lu-DOTA-^{TzAz}A33 estimated from ^{89}Zr biodistribution data (left column), ^{89}Zr-immunoPET data (center column), and ^{177}Lu biodistribution data (right column) in mice bearing subcutaneous SW1222 tumor. The data corresponds with the data shown in *Figure 5H* (*Below the limit of detection; ‡no VOI analyzed)

Mouse #	PET %ID/g	μCi /mm^3	kBq/mm^3
1	10.5	1.4	51.0
2	31.5	4.2	153.5
3	28.6	3.8	139.1
4	25.4	3.3	123.7
5	28.8	3.8	140.2
6	15.7	2.1	76.5

Table S11. Tumoral biodistribution data derived from PET images acquired 48 h after the intravenous administration of [^{89}Zr]Zr-DFO-[^{177}Lu]Lu-DOTA-^{TzAz}A33 [100 μCi (3.7 MBq) of ^{89}Zr and 200 μCi (7.4 MBq) of ^{177}Lu] to SW1222 xenografted athymic nude mice. The data corresponds with the data shown in *Figure S10*.

Mouse Number	Survival (# days)	Reason for Euthanasia
**1**	23	Tumor > 2000 mm^3
**2**	n/a	
**3**	76	Tumor > 2000 mm^3
**4**	71	Tumor > 2000 mm^3
**5**	n/a	
**6**	82	Tumor > 2000 mm^3

Table S12. Survival results for each of the mice in the longitudinal therapy study.

Supplementary Schemes

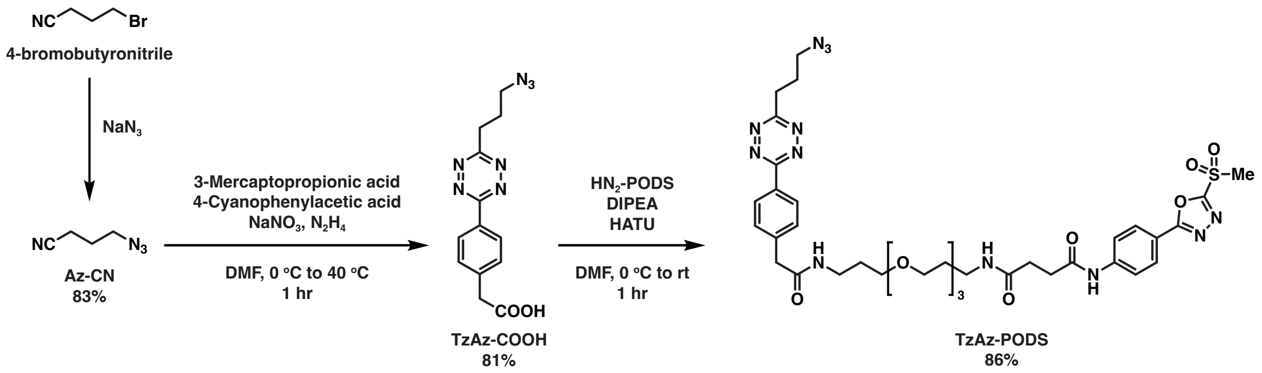

Scheme S1. The synthesis of TzAz-PODS

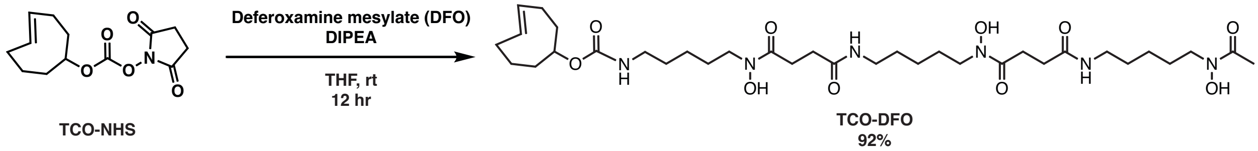

Scheme S2. The synthesis of TCO-DFO

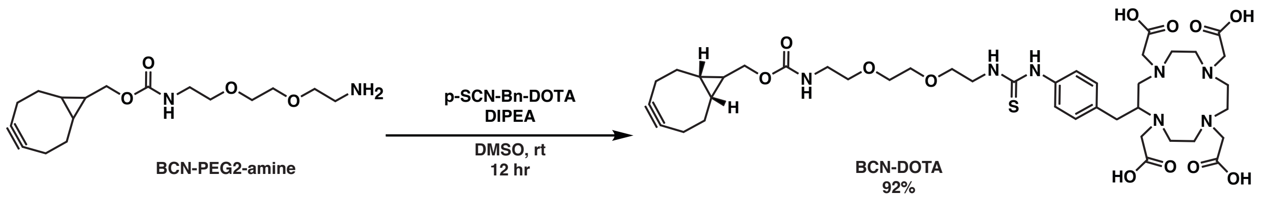

Scheme S3. The synthesis of BCN-DOTA

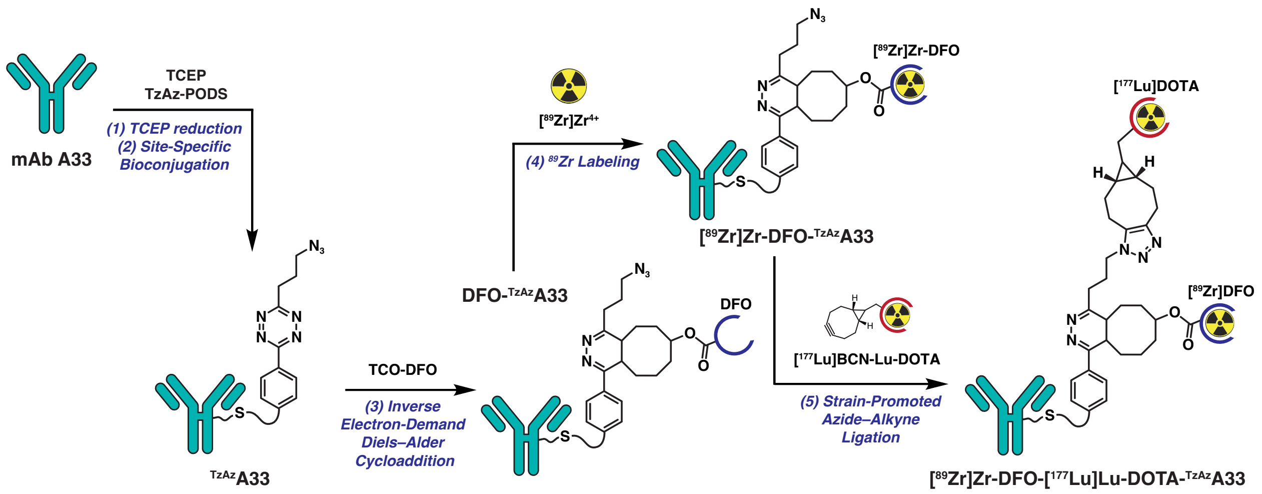

Scheme S4. The five sequential steps in the preparation of [^{89}Zr]Zr-DFO-[^{177}Lu]Lu-DOTA-^{TzAz}A33: (1) TCEP reduction, (2) site-selective bioconjugation, (3) TCO-DFO click reaction, (4) [^{89}Zr]Zr^{4+} radiolabeling, and (5) [^{177}Lu]BCN-Lu-DOTA click reaction

References

(1) Wang, Y.; Zhang, J.; Han, B.; Tan, L.; Cai, W.; Li, Y.; Su, Y.; Yu, Y.; Wang, X.; Duan, X.; Wang, H.; Shi, X.; Wang, J.; Yang, X.; Liu, T. Noncanonical Amino Acids as Doubly Bio-Orthogonal Handles for One-Pot Preparation of Protein Multiconjugates. *Nat. Commun.* **2023**, *14* (1), 974.

(2) Lyashchenko, S. K.; Esposito, T. V.; Tran, T.; Bauer, D.; Jones, K.; Park, H.; Carter, L. M.; Pillarsetty, N. V. K.; Lewis, J. S. Radiolabeling of CHX-A"-DTPA-Antibody Conjugates with [89Zr]ZrCl4. *J. Nucl. Med.* **2026**, *67* (1), 132–138.

(3) Sharma, S. K.; Adumeau, P.; Keinänen, O.; Sisodiya, V.; Sarvaiya, H.; Tchelepi, R.; Korsen, J. A.; Pourat, J.; Edwards, K. J.; Ragupathi, A.; Hamdy, O.; Saunders, L. R.; Rudin, C. M.; Poirier, J. T.; Lewis, J. S.; Zeglis, B. M. Synthesis and Comparative In Vivo Evaluation of Site-Specifically Labeled Radioimmunoconjugates for DLL3-Targeted ImmunoPET. *Bioconjug. Chem.* **2021**, *32* (7), 1255–1262.

(4) Delaney, S.; Grimaldi, C.; Houghton, J. L.; Zeglis, B. M. MIB Guides: Measuring the Immunoreactivity of Radioimmunoconjugates. *Mol. Imaging Biol.* **2024**, *26* (2), 213–221.

(5) Sharma, S. K.; Lyashchenko, S. K.; Park, H. A.; Pillarsetty, N.; Roux, Y.; Wu, J.; Poty, S.; Tully, K. M.; Poirier, J. T.; Lewis, J. S. A Rapid Bead-Based Radioligand Binding Assay for the Determination of Target-Binding Fraction and Quality Control of Radiopharmaceuticals. *Nucl. Med. Biol.* **2019**, *71*, 32–38.
